# The Global Durum Wheat Panel (GDP): An International Platform to Identify and Exchange Beneficial Alleles

**DOI:** 10.3389/fpls.2020.569905

**Published:** 2020-12-21

**Authors:** Elisabetta Mazzucotelli, Giuseppe Sciara, Anna M. Mastrangelo, Francesca Desiderio, Steven S. Xu, Justin Faris, Matthew J. Hayden, Penny J. Tricker, Hakan Ozkan, Viviana Echenique, Brian J. Steffenson, Ron Knox, Abdoul A. Niane, Sripada M. Udupa, Friedrich C. H. Longin, Daniela Marone, Giuseppe Petruzzino, Simona Corneti, Danara Ormanbekova, Curtis Pozniak, Pablo F. Roncallo, Diane Mather, Jason A. Able, Ahmed Amri, Hans Braun, Karim Ammar, Michael Baum, Luigi Cattivelli, Marco Maccaferri, Roberto Tuberosa, Filippo M. Bassi

**Affiliations:** ^1^Council for Agricultural Research and Economics-Research Centre for Genomics and Bioinformatics, Fiorenzuola d’Arda, Italy; ^2^Department of Agricultural and Food Sciences, University of Bologna, Bologna, Italy; ^3^Council for Agricultural Research and Economics-Research Centre for Cereal and Industrial Crops, Foggia, Italy; ^4^Council for Agricultural Research and Economics-Research Centre for Cereal and Industrial Crops, Bergamo, Italy; ^5^Cereal Crops Research Unit, Edward T. Schafer Agricultural Research Center, United States Department of Agriculture, Agricultural Research Service, Fargo, ND, United States; ^6^Agriculture Victoria, Agribio, Centre for AgriBiosciences, Bundoora, VIC, Australia; ^7^School of Applied Systems Biology, La Trobe University, Bundoora, VIC, Australia; ^8^School of Agriculture, Food and Wine, Faculty of Sciences, Waite Research Institute, The University of Adelaide, Adelaide, SA, Australia; ^9^Department of Field Crops, Faculty of Agriculture, Çukurova University, Adana, Turkey; ^10^Centro de Recursos Naturales Renovables de la Zona Semiárida, Departamento de Agronomía, Universidad Nacional del Sur-Consejo Nacional de Investigaciones Científicas y Técnicas, Bahía Blanca, Argentina; ^11^Department of Plant Pathology, University of Minnesota, St. Paul, MN, United States; ^12^Swift Current Research and Development Centre, Agriculture and Agri-Food Canada, Swift Current, SK, Canada; ^13^International Center for Agricultural Research in the Dry Areas, Beirut, Lebanon; ^14^State Plant Breeding Institute, University of Hohenheim, Stuttgart, Germany; ^15^Plant Sciences and Crop Development Center, University of Saskatchewan, Saskatoon, SK, Canada; ^16^International Maize and Wheat Improvement Center, Texcoco de Mora, Mexico

**Keywords:** durum wheat, genetic diversity, selection sweep, breeding history, wheat initiative

## Abstract

Representative, broad and diverse collections are a primary resource to dissect genetic diversity and meet pre-breeding and breeding goals through the identification of beneficial alleles for target traits. From 2,500 tetraploid wheat accessions obtained through an international collaborative effort, a Global Durum wheat Panel (GDP) of 1,011 genotypes was assembled that captured 94–97% of the original diversity. The GDP consists of a wide representation of *Triticum turgidum* ssp. *durum* modern germplasm and landraces, along with a selection of emmer and primitive tetraploid wheats to maximize diversity. GDP accessions were genotyped using the wheat iSelect 90K SNP array. Among modern durum accessions, breeding programs from Italy, France and Central Asia provided the highest level of genetic diversity, with only a moderate decrease in genetic diversity observed across nearly 50 years of breeding (1970–2018). Further, the breeding programs from Europe had the largest sets of unique alleles. LD was lower in the landraces (0.4 Mbp) than in modern germplasm (1.8 Mbp) at *r*^2^ = 0.5. *ADMIXTURE* analysis of modern germplasm defined a minimum of 13 distinct genetic clusters (*k*), which could be traced to the breeding program of origin. Chromosome regions putatively subjected to strong selection pressure were identified from fixation index (*F*_*st*_) and diversity reduction index (*DRI*) metrics in pairwise comparisons among decades of release and breeding programs. Clusters of putative selection sweeps (PSW) were identified as co-localized with major loci controlling phenology (*Ppd* and *Vrn*), plant height (*Rht*) and quality (gliadins and glutenins), underlining the role of the corresponding genes as driving elements in modern breeding. Public seed availability and deep genetic characterization of the GDP make this collection a unique and ideal resource to identify and map useful genetic diversity at loci of interest to any breeding program.

## Introduction

Durum wheat [*Triticum turgidum* L. ssp. *durum* (Desf.) Husn.] is the 10th most important crop worldwide with an annual production of over 40 million tons (Sall et al., 2019). It provides the raw material for semolina, pasta, couscous, burghul and several other dishes of the Mediterranean tradition (Oliveira et al., 2012). Durum wheat evolved from domesticated emmer wheat, *T. turgidum* ssp. *dicoccum* (Schrank ex Schübl.) Thell., which originated from wild emmer wheat, *T. turgidum* ssp. *dicoccoides* (Körn. ex Asch. & Graebn.) Thell. in the Fertile Crescent approximately 10,000 years ago (Ozkan et al., 2002; Dubcovsky and Dvorak, 2007). Thus, three distinct phases can be identified in the human-driven tetraploid wheat evolution process: (*i*) domestication (from wild to domesticated emmer wheat), (*ii*) continued evolution under domestication (from domesticated emmer wheat to durum wheat landraces) and (*iii*) improvements achieved by modern breeding (from landraces to modern durum wheat varieties) (Maccaferri et al., 2019). As a consequence of this evolution, four mega-germplasm groups of tetraploid wheat can be defined: tetraploid wild relatives, tetraploid primitive wheats (domesticated and cultivated), durum wheat landraces and modern durum wheat varieties. During the second evolution phase, the transition from the domesticated form of emmer to durum landraces underwent strong selection pressure by ancient farmers (Tanksley and McCouch, 1997). Modern breeding has accelerated this process by artificially crossing “best by best” and selecting for “the best” with impressive genetic gains being realized, resulting in the development of improved varieties accumulating beneficial alleles (Slafer et al., 1994; Borrelli and Trono, 2016; van Ginkel and Ortiz, 2018). Genetic gain is typically quantified as the slope of the regression between yield and year of release of varieties. A genetic gain of 0.3–1.2% per year has been recorded for durum wheat over the last century in different growing regions (e.g., Giunta et al., 2007; Royo et al., 2008; Clarke et al., 2010; Bassi and Nachit, 2019; Mondal et al., 2020) and often associated with variations in morpho-physiological traits, such as a shift toward earlier flowering and a reduced plant height, with a corresponding increase in harvest index (e.g., De Vita et al., 2007; Royo et al., 2007; Isidro et al., 2011; Bassi and Nachit, 2019). However, the positive yield trend has often been reached at the cost of eroding genetic diversity within elite gene pools (Fernie et al., 2006; Bassi and Nachit, 2019). The limited number of landraces that were used as founder lines of the modern gene pool (e.g., the first modern durum breeding program spearheaded by Nazareno Strampelli in 1910; Scarascia Mugnozza, 2005; Dexter, 2008; Royo et al., 2009; Taranto et al., 2020) and the “best × best” strategy traditionally used by breeders to drive the genetic gain (Hoisington et al., 1999; Maccaferri et al., 2003; van Ginkel and Ortiz, 2018) are the two main causes of this phenomenon. Genetic erosion of the durum wheat cultivated gene-pool in comparison with wild relatives and landraces has been reported, analogously to other crop species (Tanksley and McCouch, 1997; Gur and Zamir, 2004; Raman et al., 2010; Royo et al., 2010; Laidò et al., 2013; Kabbaj et al., 2017; Maccaferri et al., 2019), and it represents a real concern for breeders as it might lead to a lack of novel beneficial alleles for selection, yield stagnation, and/or increased susceptibility to biotic and abiotic stresses. Therefore, breeders are devoting increasing resources and effort to identify beneficial alleles and traits from novel germplasm sources to reinvigorate their programs. Indeed, pre-breeding activities have been pursued by international programs at ICARDA (Zaïm et al., 2017; Bassi et al., 2019; Robbana et al., 2019; El Haddad et al., 2020) and CIMMYT (Singh et al., 2018; Ledesma-Ramírez et al., 2019), and by national research institutes to introgress beneficial alleles from landraces and wild relatives, in parallel to international initiatives which aim to identify, collect, conserve and use the wild cousins of some of the most important food crops, as the CWR project “Adapting Agriculture to Climate Change: Collecting, Protecting and Preparing Crop Wild Relatives^1^. Population structure and genetic diversity have been studied in several modern and landrace collections of durum wheat. Many studies have focused on panels from a restricted country/area such as landraces from Southern Italy (Marzario et al., 2018), Iran (Talebi and Fayaz, 2016), Spain (Giraldo et al., 2016), Tunisia (Robbana et al., 2019; Slim et al., 2019), Turkey and Syria (Baloch et al., 2017), Palestine, Jordan and Israel (Abu-Zaitoun et al., 2018), or specific breeding programs (N’Diaye et al., 2018). Others have considered durum wheat collections of wider origin encompassing a few hundred entries. Among the earliest studies reporting on assembling international and diverse panels of mainly elite durum lines and cultivars, Maccaferri et al. (2005, 2006, 2010, 2011), Reimer et al. (2008) and Laidò et al. (2013) all reported on the genome-wide molecular diversity and LD-decay rate estimated with SSR and DArT^TM^ markers. More recently, germplasm collections have been characterized with the Illumina iSelect 90K SNP (Maccaferri et al., 2016; Mangini et al., 2018; Saccomanno et al., 2018) and subjected to GWAS for response to diseases, root morphology, canopy traits related to phenology, photosynthesis and grain yield potential (e.g., Maccaferri et al., 2010, 2016; Canè et al., 2014; Condorelli et al., 2018). Similarly, Kabbaj et al. (2017) used a mixed set of modern lines and landraces to define the genetic diversity and origin of modern durum wheat as well as to identify loci controlling resistance to insect pests and tolerance to heat stress (Bassi et al., 2019; El Hassouni et al., 2019). The largest study to date considered a collection of 429 USDA-ARS durum entries including cultivars and landraces from 64 countries. This collection was analyzed with 6,538 polymorphic SNPs (Chao et al., 2017) from the Illumina iSelect wheat 9K array (Cavanagh et al., 2013). More recently, a deeper study of genetic diversity was carried out for the Tetraploid wheat Global Collection (TGC) consisting of 1,856 single-seed purified gene bank entries chosen to comprehensively explore the diversity in tetraploid wheat from durum landraces through domesticated and wild emmer (Wang et al., 2014) in combination with the availability of the reference genome assembly of the cultivar ‘Svevo’ (Maccaferri et al., 2019).

Genetic diversity is not necessarily considered as relevant *per se*. Rather, with advances in genetics, genomics and functional genomics (Tuberosa and Pozniak, 2014), researchers and breeders are increasingly targeting specific genomic regions known to be relevant, with the objective to improve the *exploitable* and *useful* diversity (Kabbaj et al., 2017; N’Diaye et al., 2018). Accordingly, developing a detailed knowledge at the molecular level of historical loss of diversity events, together with the identification of successful allelic combinations progressively accumulated over repeated breeding cycles, are instrumental for a more effective management of breeding programs (Pfeiffer et al., 2001).

With this aim, the international durum wheat research community met in Bologna, Italy, in 2015 under the umbrella of the Expert Working Group on Durum Wheat Genomics and Breeding, as part of the Wheat Initiative^2^, to take joint action toward the identification of beneficial alleles and to make them available for breeding programs and pre-breeding efforts. The result of this international call to action is presented here under the name of the Global Durum wheat Panel (GDP). This panel was designed with the aim of capturing most of the readily *exploitable* genetic diversity, sharing it freely to facilitate research discoveries, and ultimately providing a rapid mean to exchange useful alleles worldwide. This article describes the germplasm composition and genetic structure of the GDP to provide the basic knowledge needed to support its international phenotypic characterization and exploitation.

## Materials and Methods

### Plant Materials

A total of 2,503 accessions of tetraploid wheat were obtained from 25 worldwide partners representing institutions, universities, gene banks and private companies (Supplementary Table S1), all exchanged under the Standard Material Transfer Agreement (SMTA, Noriega et al., 2019) to allow full exploitation for breeding and research. This initial set of germplasm was defined as the Durum Wheat Reference Collection (DWRC, Supplementary Table S2) and grown in the 2015–2016 season at the ICARDA experimental farm in Terbol, Lebanon. The DWRC included 1,541 *T. turgidum* ssp. *durum* modern breeding accessions (cultivars, varieties and elite lines) from 49 countries/programs, an evolutionary population set from INRA France of 180 entries (Evolutionary Pre-breeding pOpulation, EPO, David et al., 2014), 416 *T. turgidum* ssp. *durum* landraces obtained from 48 countries, and 366 wild and primitive tetraploids from 37 countries (*T. turgidum* ssp. *dicoccoides* and *dicoccum*, *turgidum*, *turanicum, polonicum, carthlicum*, respectively). Each entry was planted in two rows of 2 m in length under supplemental irrigation. Fungicide and fertilizer were provided in-season, following optimal local management practices. From each plot a single tiller was selected and tagged at flowering based on spike size, phenology and shape to be representative of most plants within the same plot. From this tiller, a leaf sample was collected for initial molecular screening. At maturity, the spike of the tagged tiller was harvested and used for advancement. In the 2016–2017 season at the same field station, 10 seeds from each spike were planted in rows of 0.5 m in length. Irrigation and chemical treatments were used to maximize productivity. Using the initial molecular data, a subset of approximately 1,000 entries were selected and defined as the Global Durum wheat Panel (GDP). The whole row was bulk-harvested and used for further advancement. In the 2017–2018 season, each entry was planted in plots of 6 m^2^ at the American University of Beirut (AUB) experimental farm in Lebanon. Fungicide, irrigation and fertilizer were applied in order to maximize productivity. Plots were visually inspected for homogeneity and off-types were manually rouged.

From this first multiplication, a total of 762 entries produced enough seed for distribution to 28 collaborators under the name of GDP version 1 (GDPv1-19), which substantially included all *T. durum* lines (modern, EPO, and landraces germplasm) (Supplementary Table S3). In the 2018–2019 season, a second and final multiplication cycle was conducted to produce enough seed of 976 entries to generate sets of 50 seeds per entry, ready to sow by 21 requesting partners. These sets were distributed under the name of GDP version 2 (GDPv2-20) (Supplementary Table S3). Unfortunately, some entries were lost during multiplication due to excessive susceptibility to yellow rust races in Lebanon. Additional sets remain available for request and distribution under SMTA at this link: http://indms.icarda.org/. Furthermore, 42 additional entries were included in GDPv2-20, mostly representing recently released European varieties and *T. durum* lines carrying introgressions of *Fhb1* developed by Boku University (Prat et al., 2017; Supplementary Table S3).

### DNA Extraction and Genotyping

The initial molecular screening of the DWRC was performed by sending one leaf from each selected tiller to LGC Genomics (United Kingdom) for DNA extraction and subsequent analyses. Ninety-four KASP^®^ markers (Supplementary Table S2) were selected because evenly distributed along the genome and highly polymorphism (Kabbaj et al., 2017), including markers tagging important loci: *PpdA1*, *VrnA1*, and *RhtB1*. Accessions with more than 50% missing data were discarded, as well as markers which were monomorphic or detected multiple loci (gene calls with multiple allelic classes and heterozygous calls at high frequency).

Lines selected to be part of the GDP were genotyped using the Illumina iSelect 90K SNP array technology (Wang et al., 2014) at the USDA-ARS Small Grain Genotyping Laboratory, Fargo, ND, United States. A pool of three seeds originating from the single spike selected in 2015–2016 were sown in Jiffy pots; 10 days old leaves were collected and DNA extracted using the NucleoSpin Plant II kit (Macherey-Nagel) according to manufacturer’s instructions. The raw data (Theta/R) from single genotyping experiments was exported from GenomeStudio software (Illumina Ltd.) and jointly analyzed for cluster assignment and genotype calling using a custom script as described in Maccaferri et al. (2019). The script parameters were *d* = 3, to call samples only within three standard deviations from a known cluster position, and *r* = 0.8, minimum confidence score that the sample belonged to the cluster to which it was assigned versus the next closest cluster. Stepwise data curation was conducted on polymorphic SNP markers. First, markers with minor alleles present in fewer than three genotypes were discarded. Second, the remaining markers were filtered to retain SNPs with a unique map position in the available genetic maps (Maccaferri et al., 2015, 2019), and with the marker sequences aligned to a single position along the Svevo reference genome RefSeq V1.0 (Maccaferri et al., 2019). Third, those markers showing multiple hits along the genome were checked for linkage disequilibrium (LD) against the hypothetical nearby mapped markers, and assigned a unique position based on the highest *r*^2^ (above a 0.3 threshold) with the putatively contiguous markers. SNP imputation was performed using *Beagle 5* software using default parameters (Browning et al., 2018). The imputation accuracy was measured at 98.6% by running 1,000 replicates of randomly masked 1% of the called genotypes (Nothnagel et al., 2009; Hancock et al., 2012). Using the software PLINK (Chang et al., 2015), redundant markers were pruned based on genome wide *linkage disequilibrium* set at *r*^2^ = 0.99 and merged into one unique SNP call. Moreover, three additional pruned hapmaps were produced selecting a single SNP among those with *r*^2^ of 0.8, 0.5 and 0.3 to run the population structure analysis.

### Genetic Diversity Within the GDP and Putative Signal of Selection Sweeps

Genetic diversity and population differentiation within the GDP, both at the genome-wide and at the single-locus level, were assessed within and between populations defined according to passport data provided by contributors or retrieved from GRIS (Genetic Resources Information System for Wheat and Triticale) through www.wheatpedigree.net. Accessions of wild emmer, primitive cultivated sub-species, and durum landraces were classified on the basis of the country of collection, whereas modern durum germplasm (cultivars, varieties and elite lines) were grouped based on the breeding program of origin and decade of release (five decades considered: ‘70–’80, ‘81–’90, ‘91–’00, ‘01–’10, and ‘11–’18). Because the year of release was not available for elite lines included in the GDP, the year in which the cross was performed was used to estimate the year of release by adding 10 years. Polymorphic SNP datasets were selected according to the set filtering for minor allele frequency (MAF) > 5% and pruning at *r*^2^ < 0.99.

Genetic diversity among and within populations was calculated by AMOVA, fixation index (*F*_*st*_, Wright, 1965) and the polymorphism information content (PIC, Botstein et al., 1980). The within populations total number of polymorphic loci (N), Nei’s gene diversity (Nei, 1973), and mean number of pairwise differences were calculated, and significance was determined based on LSD at *P* < 0.05. Population differentiation was assessed based on Nei’s genetic distance (Nei, 1972) and population pairwise *F*_*st*_. All values were derived using the Arlequin 3.5 software (Excoffier and Lischer, 2010), and significance levels for variance components and *F*_*st*_ statistics were estimated based on 10,000 and 1,000 permutations, respectively.

Furthermore, single locus analyses of genetic diversity across the whole genome were conducted to identify genomic regions putatively affected by human-driven selection sweeps. Signals of putative selection sweeps were assessed using a hapmap pruned for *r*^2^ < 0.99 calculating two different indices: *F*_*st*_ was estimated by Arlequin 3.5 software, and the diversity reduction index (*DRI*) was calculated using the modified *ROD* formula presented in Maccaferri et al. (2019). To reduce spurious signals due to different coalescence time between SNPs, the raw single SNP-based results were smoothed by averaging with a sliding window of 15 SNPs with a one-marker step. Significance of selection signals was assessed in a two-step procedure. In the first step, signal peaks falling in the top 10% percentile of the distribution were identified. Additional neighboring signals were merged into the one representing the highest value, considering as neighbors loci falling within a physical distance lower than the LD. After merging adjacent peaks, the index distribution (Jordan et al., 2015) was re-calculated and the 95th percentile was chosen as the index-specific significance threshold.

### Population Structure Analysis and Selection of the GDP Collection

A preliminary population stratification analysis was carried out on the DWRC panel using a curated set of 88 KASP^(R)^ markers. The GDP set was then re-stratified using the Illumina 90k SNP genotyping data and three possible pruned hap-maps (*r*^2^ set at 0.3, 0.5, and 0.8) were considered in order to optimize the trade-off between uniformity of genomic sampling and informativeness. Based on the analysis results, the pruned SNP-set at *r*^2^ = 0.5 was used for all subsequent population structure analyses. For both the DWRC and GDP, the population structure was estimated by the model-based likelihood method *ADMIXTURE* optimized using the block relaxation algorithm and the quasi-Newton convergence acceleration method and *q* = 3 secants (Alexander et al., 2009), as well as by means of Ward’s clustering of Nei’s genetic distances, using the *poppr* v. 2.8.3 and *adegenet* packages of R (Jombart, 2008; Kamvar et al., 2014; R Core Team, 2016). For both methods, the sub-population membership was defined for *k* values increasing from 2 to 20. The parameters used to define the optimal number of clusters were *ADMIXTURE*’s cross-validated error rate and minimum group size. Lines with strong admixture were defined as those showing less than 30% identity (membership) with any ancestry in the model-based likelihood analysis. Because the GDP is a selected sub-set of the initial DWRC panel, the population stratification was first used to define the most representative DWRC entries to be included in the GDP, and secondly to define what degree of genetic diversity was lost because of the sub-sampling process. Pairwise similarity estimated as identity-by-state (IBS) was also calculated for the DWRC population to filter for duplicated/highly similar entries using TASSEL5 software (Bradbury et al., 2007). To select the subset of DWRC entries that composed the GDP the following procedures were followed. First, genotypes representing historical founders, parents of mapping populations, or known germplasm carrying interesting alleles/phenotypic traits were included, while the name and pedigree were inspected and compared to the similarities defined at the molecular level (IBS-GS matrix) to discard duplicated entries with >0.95 similarity (only one entry was retained per group). The remaining entries were classified into six groups, five of which were defined by genetic structure at *k* of 5, and one extra split to incorporate the EPO set, which was clearly differentiated from the other groups. The GDP collection was then assembled through a *stratified-sampling method*, therefore choosing representative entries from each main Ward’s cluster and sub-clusters, depending on each subgroup/subspecies being considered and chosen in order to maximize the number of sub-clusters being considered for GDP sampling. Genotypes with low average genetic similarity to other entries (rare haplotypes) were also chosen. The genetic diversity level present in the two collections was compared to confirm that no major genetic diversity losses occurred after sampling the GDP from the DWRC. The Shannon-Wiener’s diversity index, Nei’s expected heterozygosity, allelic evenness (Shannon, 1948; Nei, 1978; Smith and Wilson, 1996), MAF, and the site frequency spectrum (SFS) distribution were assessed at the *locus* level both in the DWRC and GDP based on the 88 KASP markers. Diversity indexes analyses were conducted using the “*locus_table*” and “*poppr*” function of the *poppr R* package (Kamvar et al., 2014).

### LD Decay

Pairwise marker correlations (*r*^2^ values) were calculated on the SNP dataset of the GDP for each chromosome using TASSEL5 (Bradbury et al., 2007). LD decay curves were fitted using the non-linear model described in Rexroad and Vallejo (2009). Critical parameters of marker distances at *r*^2^ = 0.3 and 0.5 were extrapolated from the fitted regression curves. The *r*^2^ of unlinked markers (background noise) was estimated as the 95th quantile of *r*^2^ values of markers on different chromosomes (unlinked set). To estimate the local LD value along chromosomes, each marker LD was calculated using the mean *r*^2^ with the 50 nearest markers, and then smoothed as one value using the step-sliding window.

### Identification and Clustering of Putative Selection Sweep (PSW) Signals

Detection of putative selection sweep (PSW) signals was based on genome-wide *F*_*st*_ and *DRI* metrics calculated for *modern* vs. *landraces* and for pairwise groups of entries classified by decade or breeding program. PSW clusters were defined as two significant signals on the same chromosomal region in a single pair/comparison or among pairs/comparisons. Moreover, signals also partially overlapping were grouped into one cluster. The catalog of PSW was integrated with data from the literature that included major genes cloned in wheat, known QTL and the comprehensive catalog (a.k.a. *QTLome*) defined in Maccaferri et al. (2019).

## Results

### From the Durum Wheat Reference Collection to the Global Durum Wheat Panel

The original DWRC was comprised of 2,503 accessions that were genotyped with 94 KASP^(R)^ markers (Supplementary Table S2). The curation process yielded a final set of 2,493 accessions (99.1%), each with 88 (93.6%) reliable KASP^(R)^ marker profiles. Population structure assessed by *ADMIXTURE* (Supplementary Figure S1) highlighted three subsets at *k* = 3: (*i*) a group including *T. turgidum* spp. *dicoccum* and *dicoccoides*, (*ii*) a second group including modern durum wheat germplasm and (*iii*) a third group comprising modern North American germplasm together with most durum landraces and accessions of the primitives *T. turgidum* spp. *turgidum*, *turanicum* and *polonicum* as durum-related sub-species. At *k* = 4 the North American modern germplasm was separated from landraces and the mentioned primitive subspecies. Finally, at *k* = 5 the group of the modern durum wheat germplasm was further subdivided in two groups: the first one tracing its ancestry to the CIMMYT breeding program, and the second one composed of the Southern European germplasm and those entries with ancestry from the ICARDA breeding program. The structure of the population was confirmed using bootstrapped Ward’s clustering (Supplementary Figure S2).

**FIGURE 1:**
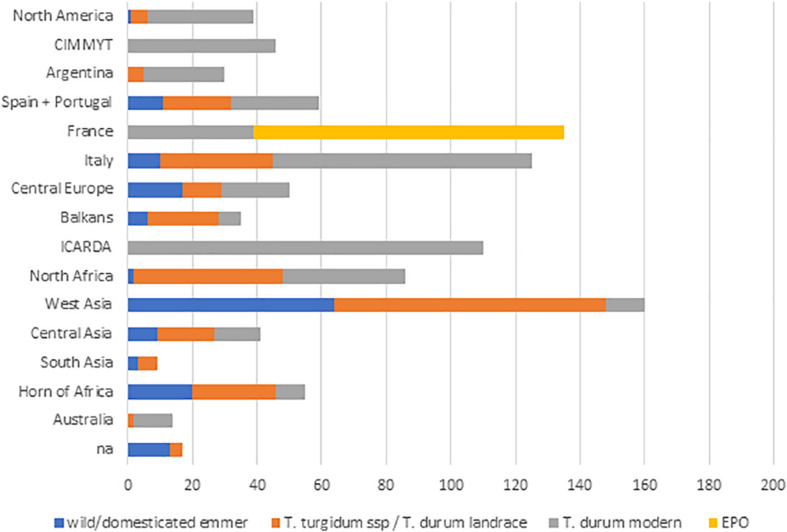
Distribution of the geographic origin of the GDP accessions used for genetic diversity analysis. Countries of origin are grouped as follows: *Central Europe*: Austria, Hungary, Ukraine, Sweden, Poland, United Kingdom, and Germany; *Balkans*: Serbia, Bosnia, and Herzegovina, Bulgaria, Romania, Greece, and Crete; *North Africa*: Egypt, Libya, Algeria, Tunisia, and Morocco; *West Asia*: Turkey, Syria, Lebanon, Israel, Jordan, Iran, Iraq, Armenia, Azerbaijan, Georgia, Oman, Yemen, and Saudi Arabia; *Central Asia*: Kazakhstan, Afghanistan, Russia, Uzbekistan, and China; *Horn of Africa*: Ethiopia, Eritrea, and Kenya.

A total of 398 genotypes represented identical entries contributed by multiple partners. The remaining entries were divided into six groups: five defined by genetic structure at *k* = 5 and one additional group to incorporate the EPO set. When each of these subsets was subjected to population structure assessment based on Ward’s clustering, the sub-clustering concurred with the clustering computed on the whole DWRC and a detailed picture of group differentiation based on geographic origin was revealed. The entries to be included in the GDP were then identified based on the Ward’s clustering using a *stratified-sampling method*. Following the criteria defined in Material and Methods, three groups of durum wheat modern germplasm were selected (Supplementary Figure S3): (*i*) CIMMYT- and ICARDA-derived genetic materials, and modern semi-dwarf and vernalization-insensitive lines mostly adapted to the Mediterranean environment for a total of 288 genotypes; (*ii*) 96 elite semi-dwarf durum wheat lines with photoperiod and/or vernalization sensitivity mainly developed in Canada, France, Italy, and Central Europe; (*iii*) 96 non-semi-dwarf durum wheat lines of different origins. Three additional groups were selected to incorporate more genetic diversity including; (*iv*) 96 EPO lines (Supplementary Figure S4); (*v*) 192 durum wheat landraces representing the geographical distribution of the original collection (Supplementary Figure S5); and (*vi*) a final group including domesticated emmer lines (96, Supplementary Figure S6), wild emmer accessions and other tetraploid primitives (96, Supplementary Figures S7, S8, respectively). A seventh group of 42 entries including recently registered European varieties and durum lines carrying *Fhb1* introgressions developed at the Boku University (Austria) was also included. The final GDP selection consisted of 1,028 accessions, 976 of which were multiplied in sufficient quantity and quality for seed re-distribution by ICARDA, while 42 among European varieties and accessions with *Fhb1* introgressions are available from University of Bologna and Boku University, respectively (Supplementary Table S3) for a total of 1,018 entries available as seed stocks. Figure 1 shows the geographic origin of the GDP accessions.

To assess the extent of the genetic diversity loss in the sampling process from the DWRC to GDP, different indices were calculated based on the KASP data for the two panels. Locus level correlations between DWRC and GDP values resulted in Pearson’s coefficients of 0.94 for the MAF, 0.95 for allelic evenness, 0.96 for expected heterozygosity and 0.97 for Shannon-Wiener’s diversity index (Supplementary Figure S9), indicating that the sampling process that originated the GDP caused a 3–6% loss of the initial DWRC diversity. The SFS (Supplementary Figure S10) showed that the distribution of the allele frequencies in the GDP is comparable to that observed in the initial DWRC, except for an appreciable decrease in three rare allele frequency classes (MAF: 0.05–0.10, 0.10–0.15, and 0.35–0.40) and a corresponding increase for three high frequency classes (MAF: 0.15–0.20, 0.30–0.35, and 0.45–0.50).

### Deep Genotyping of the Global Durum Wheat Panel (GDP)

Genotyping of the GDP with the iSelect 90K wheat SNP array generated 42,520 polymorphic SNPs. After several quality filtering steps, a total of 16,633 SNP markers were retained and imputed for missing data. Both datasets are available at the repositories GrainGenes^3^ and T3/Wheat^4^. The tetraploid genome was thus probed by a mean of 1,188 SNP markers per chromosome with an average density of 1.7 SNPs per Mbp or 6.3 SNPs per cM (Table 1). Almost one third (4,119) of the consecutive SNPs were located within 0.5 Kbp of each other, possibly due to the redundancy of the Illumina 90K SNP design, and 4,938 SNPs were located at various interlocus distances between 1 and 100 Kbp. The remaining 7,259 SNPs mapped at distances from >0.1 to 5 Mbp, and only 302 SNPs mapped at distances >5 Mbp (Supplementary Figure S11A). The genome coverage calculated as a percent of the physical genome length probed by SNP markers was almost complete with an average of 0.998% (Table 1). The marker density along the chromosomes was higher in proximal and distal portions compared to pericentromeric regions (Supplementary Figure S11B), and the opposite for the interlocus distances (Supplementary Figure S11C).

**TABLE 1:** Genome coverage by the SNP marker dataset expressed for each chromosome and genome.

Chromosome	N° SNP	Mean SNP/Mb	Chromosome coverage (%)
1A	1,138	1.9	0.998
1B	1,638	2.4	0.997
2A	1,096	1.4	0.999
2B	1,800	2.3	0.997
3A	979	1.3	0.999
3B	1,250	1.5	0.999
4A	750	1.0	0.996
4B	863	1.3	0.998
5A	962	1.4	0.997
5B	1,419	2.0	0.999
6A	974	1.6	0.998
6B	1,267	1.8	0.993
7A	1,248	1.7	0.998
7B	1,249	1.7	0.997
Genome A	7,147	1.5	0.998
Genome B	9,486	1.9	0.997
*Total*	*16,633*	*1.7*	*0.997*
*Mean*	*1,188*	*1.7*	*0.998*

After excluding six accessions due to failed genotyping, filtering carried out at the accession level based on IBS_GS matrix (Supplementary Table S4) allowed for the identification of 10 accessions whose genotypic data were not relevant (misclassified accessions or contaminated DNA) that were discarded from further analysis. High-density genotyping data are therefore available for a final set of 1,011 accessions, while for a total of 1001 accessions both seed stock and genotypic data are provided (Supplementary Table S3).

### Genetic Diversity Analysis

Genotyping data allowed to characterize the GDP for genetic diversity and differentiation within and among groups defined on the base of passport data (Supplementary Table S3). GDP entries were classified according to the following criteria. The introduction of the semi-dwarf *RhtB1b* allele from CIMMYT durum lines (Motzo and Giunta, 2007; Ortiz et al., 2007) represents the origin of the post green revolution germplasm, so all entries generated from crosses carried out after 1970 were considered as *modern* germplasm. North American varieties and breeding materials released after 1970 were also included in the *modern* set, even though these did not carry the *RhtB1b* allele, which is not beneficial in the northern semi-arid prairie environment. All durum lines pre-dating 1970 were considered as *landraces*, although in a few cases these were obtained through breeding selection of populations or voluntary hybridization among landraces. Notably, the characterization of genetic diversity could not clearly distinguish *T. turgidum* spp. *durum* landraces from other *T. turgidum* sub-species related to durum like *T. turgidum* ssp. *turgidum*, *turanicum* and *polonicum* (Maccaferri et al., 2019). Therefore, the genetic diversity analyses reported hereafter were carried out including all durum- related *T. turgidum* sub-species accessions as *landraces* and grouped according to the country of origin. The *EPO population* was considered as a separate group based on its highly distinct genetic structure.

**FIGURE 2:**
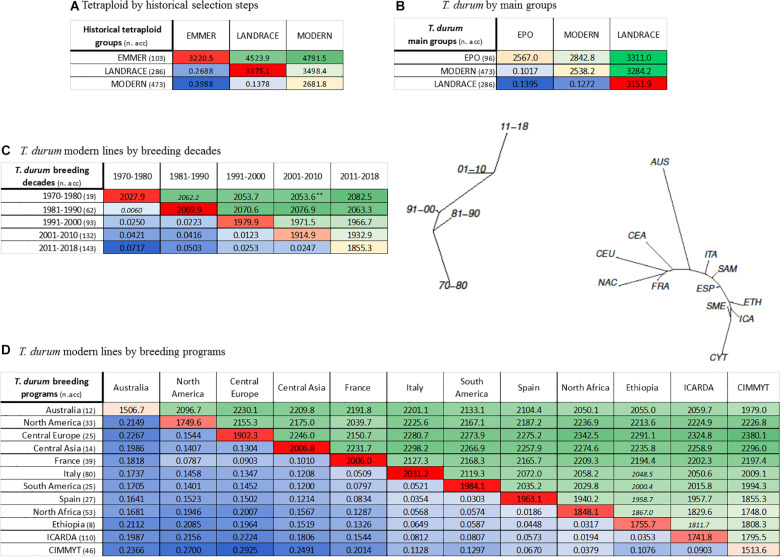
Population differentiation calculated as pairwise *F*_*st*_ and average number of pairwise differences between groups/populations defined according to passport data for: **(A)** evolution from domesticated emmer, to landraces, to modern lines; **(B)** all *T. durum* groups of EPO, landraces, modern lines; **(C)** *T. durum* modern lines classified according to decade of release; **(D)** *T. durum* modern lines classified based on breeding program. In each matrix, above diagonal elements (shades of green) contain the average number of pairwise differences, while below diagonal elements (shades of blue) report pairwise *F*_*st*_ values. Diagonal elements (shades of red) contain gene diversity within groups calculated as mean number of pairwise differences. Significance was assessed upon 1000 permutations. All values are significant at *p* < 0.001, except values marked with ** which were significant at *p* < 0.01, or values in italics that were not significant. Relative Neighbor-Joining phylogenetic tree based on Nei’s distance are also reported for panels **(C,D)**.

The primary objective was to describe the pattern of genetic diversity across the history of durum wheat evolution and breeding so these groups composed as above described were considered: (*i*) *modern* germplasm, (*ii*) *landraces* and (*iii*) emmer (*T. turgidum* ssp. *dicoccum*) accessions, for a total of 861 genotypes. AMOVA highlighted a moderate level (23%) of genetic variance distinguishing the three groups (Table 2A), with a larger portion still existing within groups (77%). Reduction of overall diversity was observed in *modern* lines with respect to both *T. turgidum* ssp. *dicoccum* and *landraces*. Durum *landraces* showed a level of genetic diversity even higher than that of *T. turgidum* ssp. *dicoccum* accessions included in the GDP, perhaps due to ascertainment bias associated to the type of genotyping array used for the analysis, originally developed to maximize polymorphism among modern bread and durum breeding lines. However, in pairwise differentiation analysis *F*_*st*_ value was higher in the comparison *landraces* vs. *dicoccum* (*F*_*st*_ = 0.2688) with respect to the comparison *landraces* vs. *modern* lines (*F*_*st*_ = 0.1378) (Figure 2A). The EPO population, which was bred by INRA based on a composite cross to introduce diversity from wild and primitive accessions of *T. turgidum* subspecies, showed a relatively high level of diversity (David et al., 2014). Considering the *all durum* dataset (885 entries and 8,802 polymorphic SNPs), AMOVA results across the three main groups (*modern* lines, *landraces* and EPO accessions) showed that the highest proportion of molecular variance (86.94%) was observed within clusters rather than among clusters (13.06%) (Table 2B). *Landraces* showed the highest value of Nei’s genetic diversity (0.358), followed by *modern* germplasm (0.292) and EPO (0.288) (Table 2B). As to among-population comparisons, the highest differentiation was found for *landrace* vs. *modern* comparisons (*F*_*st*_ = 0.127), while an *F*_*st*_ of 0.1 was calculated for the EPO vs. *modern* comparison (Figure 2B). This result is also confirmed by comparable values of PIC and *F*_*st*_ calculated for *landraces* (0.282 and 0.101, respectively, Table 2C) and *modern* lines (0.278 and 0.117, respectively, Table 2E).

**TABLE 2:** AMOVA and gene diversity for five germplasm sub-sets defined according to passport data: **(A)** GDP without the wild accessions, with grouping based on historical selection steps: *T. dicoccum* accessions, *T. durum* germplasm sub-sets landraces, *T. durum* germplasm sub-sets cultivars; **(B)** all *T. durum* germplasm sub-sets; groups are EPO, *T. durum* germplasm sub-sets landraces, modern lines; **(C)** all landraces grouped according to country of origin; **(D)** all *T. durum* germplasm sub-sets modern lines, classified according to decade of release; **(E)** all *T. durum* germplasm sub-sets modern lines, classified based on breeding program.

(A)						

Source of variation	d.f.	Sum of squares	Variance components	Percentage of variation		
Among populations	2	222,822.13	443.9	22.98		
Within populations	859	1,278,090.16	1,487.88	77.02		
*Total*	861	1,500,912.3	1,931.79			
*F*_*st*_		0.23				

***T. durum* groups**	**N° accessions**	**N° polymorphic loci over 10173**	**Nei’s gene diversity**	**Mean number of pairwise differences**		

LANDRACE	286	10,154	0.332	3,375.08		
EMMER	103	9,901	0.317	3,220.49		
MODERN	473	10,010	0.264	2,681.76		
*Mean value*			0.304	3,092.45		
*Lsd (p* = *0.0005)*			0.002	17.7		

**(B)**						

**Source of variation**	**d.f.**	**Sum of squares**	**Variance components**	**Percentage of variation**		

Among populations	2	103,704.61	207.33	13.06		
Within populations	852	1,175,524.24	1,379.72	86.94		
*Total*	854	1,279,228.85	1,587.05			
*F*_*st*_		0.13				
PIC		0.273	range (0.09–0.375)			

***T. durum* groups**	**N° accessions**	**N° polymorphic loci over 8802**	**Nei’s gene diversity**	**Mean number of pairwise differences**		

LANDRACE	286	8,796	0.358	3,151.85		
MODERN	473	8,781	0.292	2,567.05		
EPO	96	8,213	0.288	2,538.16		
*Mean value*			0.313	2,752.35		
*Lsd (p* = *0.0005)*			0.002	17.6		

**(C)**						

**Source of variation**	**d.f.**	**Sum of squares**	**Variance components**	**Percentage of variation**		

Among populations	13	62,147.3	169.12	10.1		
Within populations	270	403,266.13	1,504.72	89.9		
*Total*	281	465,413.43	1,673.84			
*F*_*st*_		0.101				
PIC		0.282	range (0.09–0.375)			

**Landrace group**	**N° accessions**	**N° polymorphic loci over 9414**	**Nei’s gene diversity**	**Mean number of pairwise differences**		

Turkey-Transcaucasian	29	9,253	0.374	3,521.67		
Central Asia	18	9,035	0.356	3,348.15		
Arabian Peninsula	9	7,790	0.349	3,285.50		
Iberian Peninsula	21	9,048	0.346	3,253.83		
Central Europe	18	8,547	0.341	3,206.41		
South Asia	6	6,640	0.329	3,094.53		
Greece	16	8,539	0.327	3,082.52		
Italy	34	8,656	0.303	2,874.58		
Ethiopia	26	8,174	0.302	2,843.20		
North Africa	47	9,059	0.301	2,839.57		
Argentina	5	6,107	0.300	2,829.40		
Levant	46	8,496	0.289	2,722.53		
North America	5	5,340	0.280	2,640.00		
Australia	2	3,136	0.333	3,136.00		
*Mean value*			0.323	3,048.42		
Lsd* (p = *0.05)*			0.005	48.1		
Lsd* (p = *0.001)*			0.008	75.8		

**(D)**						

**Source of variation**	**d.f.**	**Sum of squares**	**Variance components**	**Percentage of variation**		

Among populations	4	13,737.04	29.36	2.95		
Within populations	444	429,609.05	967.59	97.05		
*Total*	448	443,346.08	996.95			
*F*_*st*_		0.029				

**Breeding decade**	**N° lines**	**N° polymorphic loci over 5685**	**Nei’s gene diversity**	**Mean number of pairwise differences**		

70–80	19	5,334	0.357	2,027.88		
81–90	62	5,668	0.364	2,069.90		
91–00	93	5,679	0.348	1,979.88		
01–10	132	5,681	0.337	1,914.88		
11–18	143	5,675	0.326	1,855.32		
*Mean value*			0.346	1,969.57		
*Lsd (p* = *0.0005)*			0.003	33.2		

**Breeding group**	**70–80**	**81–90**	**91–00**	**01–10**	**11–18**	**Total**

Australia	0	1	2	5	4	12
Central Asia	2	1	3	2	1	9
Central Europe	0	1	4	2	12	19
CIMMYT	3	2	5	6	29	45
Spain	3	4	10	6	1	24
Ethiopia	0	0	1	1	3	5
France	0	7	12	13	7	39
ICARDA	0	8	12	45	40	105
Italy	9	12	22	23	14	80
North America	2	8	13	5	5	33
South America	0	7	1	7	10	25
South Mediterranean	0	11	8	17	17	53
Total	19	62	93	132	143	

**(E)**						

**Source of variation**	**d.f.**	**Sum of squares**	**Variance components**	**Percentage of variation**		

Among populations	11	60,189.98	121.56	11.67		
Within populations	460	423,162.29	919.92	88.33		
*Total*	471	483,352.27	1,041.48			
*F*_*st*_		0.117				
PIC		0.278	range (0.09–0.375)			

**Breeding group**	**N° lines**	**N° polymorphic loci over 5918**	**Nei’s gene diversity**	**Mean number of pairwise differences**		

Italy	80	5,885	0.343	2,031.16		
Central Asia	14	5,463	0.339	2,006.78		
France	39	5,768	0.339	2,006.03		
South America	25	5,535	0.335	1,984.11		
Spain	27	5,591	0.332	1,963.08		
Central Europe	25	5,466	0.321	1,902.33		
South Mediterranean	53	5,776	0.312	1,848.12		
Ethiopia	8	4,253	0.297	1,755.71		
North America	33	5,316	0.296	1,749.61		
ICARDA	110	5,813	0.294	1,741.78		
CIMMYT	46	5,046	0.256	1,513.59		
Australia	12	4,208	0.255	1,506.68		
*Mean value*			0.310	1,834.08		
*Lsd (p* = *0.05)*			0.005	29.7		
*Lsd (p* = *0.001)*			0.008	46.9		

*Geographic area of origin has been assigned to GDP landraces based on country where they have been sampled, as follows: North America: Canada + United States; Arabian Peninsula: Oman, Yemen, and Saudi Arabia; Central Europe: Bulgaria, Serbia, Poland, Ukraine, Hungary, Romania, United Kingdom, Germany, Sweden, Bosnia, and Herzegovina; Iberian peninsula: Spain and Portugal; Levantine: Iran, Iraq, Jordan, Syria, and Israel; North Africa: Egypt, Tunisia, Algeria, Morocco, Libya, and Malt; Turkey-Transcaucasia: Turkey, Armenia, Azerbaijan, and Georgia; South Asia: Pakistan and India; Central Asia: Kazakhstan, Russia, and Afghanistan. GDP modern lines have been assigned to breeding program based on the country where lines have been developed, as follows: South Mediterranean: Egypt, Algeria, Tunisia, Syria, Lebanon, Jordan, and Morocco; North America: Canada + United States (including Desert durum); Central Europe: Austria, Serbia, Hungary, Ukraine, and Bulgaria; Central Asia: Kazakhstan and Uzbekistan. For each summary, the first table reports results of AMOVA, the second table contains computations about gene diversity within groups/populations. AMOVA was significant at p < 10^–6^ upon 10,000 permutations. LSD for within group gene diversity was calculated at the indicated p-value considering n = the least number of group genotypes, except for the landrace group where n = 5 was chosen. For decade of release, a third table reports the composition of each decade group with respect to the breeding programs.*

Durum *landraces* (282) were grouped into 14 sub-populations according to the country of origin. This clustering process accounted only for 10.1% of the variance, while the vast majority of diversity still remained unclustered within sub-populations (Table 2C). Nei’s gene diversity values ranged from 0.280 (United States–Canada) to 0.374 (Turkey–Transcaucasian).

To analyze the changes in diversity within the *modern* germplasm over time and across breeding groups, the totality of 473 cultivars and elite lines were divided into sub-groups based on two different criteria: (*i*) decade of release from 1970 to 2018; and (*ii*) country of registration/release, which roughly defines the main groups of breeding programs. Thus, five decades (‘70–’80, ‘81–’90, ‘91–’00, ‘01–’10, ‘11–’18) and 12 breeding program groups (Australia, North America, Central Europe, Central Asia, France, Italy, South America, Spain, South Mediterranean, Ethiopia, ICARDA, CIMMYT) (Supplementary Table S3) were considered. For temporal groups (decades), AMOVA analysis revealed a very low, even if statistically significant, percentage of variation among groups (2.95%, Table 2D), attributing the near totality of variance to individuals within groups. Nei’s gene diversity showed a constant decreasing trend starting from the decade (‘81–’90) to the most recently released (2011–2018), with limited but significant variation. The mean number of pairwise differences within a decade (Figure 2C), and pairwise *F*_*st*_ among groups confirmed the trend; the highest difference in *F*_*st*_ values was observed in the comparison between the ‘70–’80s and the 2011–2018 decades, confirming a progressive and generalized shift toward the enrichment of fewer successful haplotypes during breeding history (Figure 2C).

The last analysis considered the *modern* germplasm, clustered according to breeding groups. AMOVA attributed the highest proportion of molecular variance (88.33%, Table 2E) to individuals within breeding programs, while variation between populations accounted for the remaining portion (11.67%). Moderate levels of diversity were observed for Australia and CIMMYT showing the lowest values (0.255 and 0.256, respectively), followed by ICARDA (0.294), North America (0.296), and Ethiopia (0.297), up to highest values calculated for Italy (0.343), Central Asia and France (0.339), and South America (0.335) (Table 2E). As for among-population comparisons, the Italian *modern* group showed generally lower pairwise *F*_*st*_ values as compared to all the other groups, with relatively higher values against the Northern programs and lower values against the other Mediterranean groups (Figure 2D). A reverse pattern of differentiation was evident for the French breeding programs, showing stronger similarities with the Northern programs. Low *F*_*st*_ values were calculated for pairwise comparisons among Central Europe, North America and Central Asia programs. Likewise, both CIMMYT and ICARDA showed the highest *F*_*st*_ values in the comparison with these breeding groups and the lowest *F*_*st*_ values with the Mediterranean groups. Between them, ICARDA and CYMMIT showed a *F*_*st*_ = 0.09. Analogously, low *F*_*st*_ values evidenced known interactions of international breeding programs with national programs, like ICARDA vs. Ethiopia and North African countries. The Australian breeding program appeared to stand as a separate group.

### LD Decay

**FIGURE 3:**
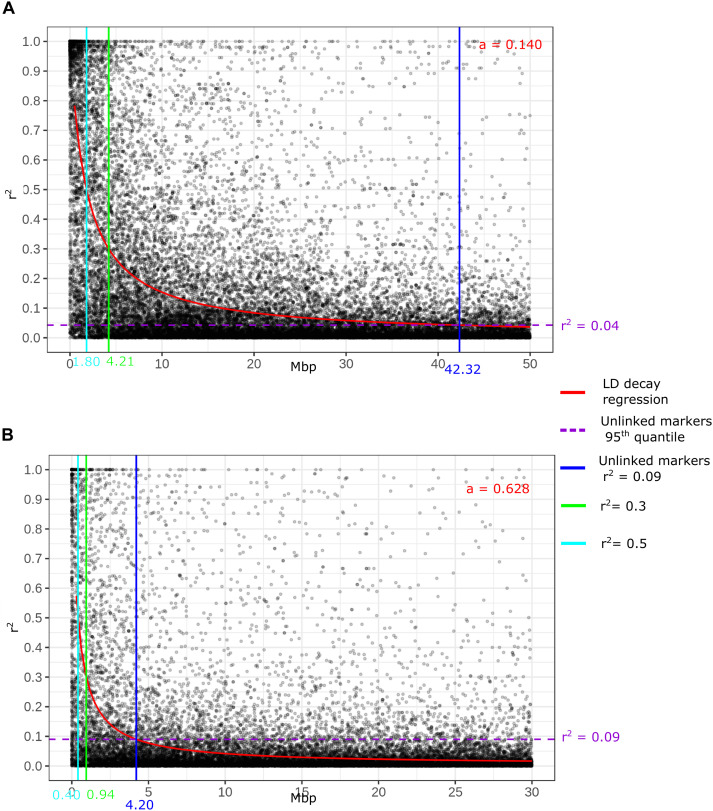
Genome wide linkage disequilibrium (LD) decay in respect to physical distance in the two main groups of the GDP collection: **(A)** *modern* germplasm, **(B)** *landraces*.

Genome-wide LD decay was calculated for the two major *T. turgidum* ssp. *durum* groups of the GDP collection: *modern* and *landraces*. As expected, LD was lower in *landraces* than in *modern* lines (Figure 3). The critical *r*^2^ values of 0.3 and 0.5 were reached at a distance of 0.9–0.4 Mbp in *landraces*, and at distances of 4.2–1.8 Mbp in *modern*. Overall, 95% of unlinked markers showed a *r*^2^ value <0.09 in *landraces* and 0.04 in *modern*. These *r*^2^ values corresponded to distances of 4.2 Mbp in *landraces* and of 42.3 Mbp in *modern*. Supplementary Figure S12 reports LD calculated for each chromosome and for *modern* and *landraces*, independently.

### Detection of Putative Selection Signals in Durum Wheat Groups

Considering the durum *modern* germplasm and its whole MAF-unfiltered SNP dataset of 16,633 SNPs, 889 *unique* breeding program-specific alleles were found (5.4% of the total, Supplementary Table S5). “Unique” is used to define a minor allele that occurs only in the germplasm of one breeding program and not in any other. The groups with the largest set of unique alleles were Central Europe, Central Asia, and Italy, with 289, 208, and 102 unique alleles, respectively (Table 3). Ethiopia and Australia were characterized by the lowest number of unique alleles with 13 and 9, respectively. It was then possible to identify rare alleles (with MAF less than 0.05) within the group of unique alleles. In particular, *rare unique alleles* were observed in all of the breeding groups except Australia, South America, and Ethiopia, ranging between 39 and 100% of the unique alleles. It was interesting to note that for CIMMYT and ICARDA, 100% of unique alleles were also rare, similarly to Italy (99%). Among the remaining unique alleles, none was a frequent allele in the target breeding group, and most (64%) had frequency from 5 to 10%. However, 53 SNPs showed higher frequency, suggesting a role in a specific breeding target or for adaptation to the corresponding environmental conditions.

**TABLE 3:** Unique alleles in the different breeding groups.

Breeding groups	N° of unique alleles	N° of unique alleles with MAF > 5% in the target group	Average frequency of the unique alleles in the target group
Central Europe	289	122	0.11
Central Asia	208	208	0.07
Italy	102	1	0.03
ICARDA	60	0	0.01
North Africa	57	2	0.02
North America	49	9	0.04
France	46	9	0.03
South America	23	14	0.09
Spain	22	5	0.05
CIMMYT	21	0	0.02
Ethiopia	13	13	0.14
Australia	9	9	0.16
*Tot*	899	392	0.06

Fixation of loci controlling traits of interest by intense selection during the breeding process may result in steep increases in allele frequency, reduced variation (reported as a *selective sweep*), and therefore divergence in allele frequency in the proximity of the selected loci. Low-resolution genomic scans can be used to identify regions containing loci and causative genes with a putative major influence on breeding processes. Scans for PSW between *modern* and *landraces* (Supplementary Table S6) identified 53 PSW clusters, based on *F*_*st*_ only (24) or on both indices, *F*_*st*_ and DRI (8). Most clusters (73%) extended for less than 50 Mbp, but three extended for >150 Mbp. All chromosomes were found to carry PSW clusters, with chromosome 1B being the most targeted by breeders’ selection. Promising putative candidate genes were found to co-locate with eleven PSW clusters, for instance the genes *Rht1-B* and *Ppd-A1* on chromosomes 4B and 2A, respectively (Supplementary Table S6). Considering four subsequent decades of release, 62 putative signal clusters were highlighted across all six pairwise comparisons between the four decades (Supplementary Table S7). Chromosome 2B showed the highest number (9) of PSW clusters, whereas only two clusters per chromosome were identified on chromosomes 4A, 4B and 5B. Considering the five decades comparisons separately, 92 putative signals were found for *DRI*, 74 for *F*_*st*_, and 46 were confirmed by both methods. The signals were distributed across the four comparisons: 30 were found for the ‘70–’80 vs. ‘81–’90 decades, 33 for both the comparisons ‘81–’90 vs. ‘91–’00 and ‘91–’00 vs. ‘01–’10, and 24 for ‘01–’10 vs. ‘11–’18. Most clusters were identified for two different decade comparisons (32, 10, and 2 PSW clusters, respectively), while 18 PSW clusters were detected in a single comparison. PSW clusters physical size extended from 11 Mbp for cluster *Cls-chr3B.1* to 386 Mbp for Cls-*chr6A*.*4*, with an average of 52 Mbp (Supplementary Table S7). As expected, the largest clusters were predominantly located in centromeric and peri-centromeric regions. Promising putative candidate genes were found to co-locate with nine PSW clusters (Supplementary Table S7).

**FIGURE 4:**
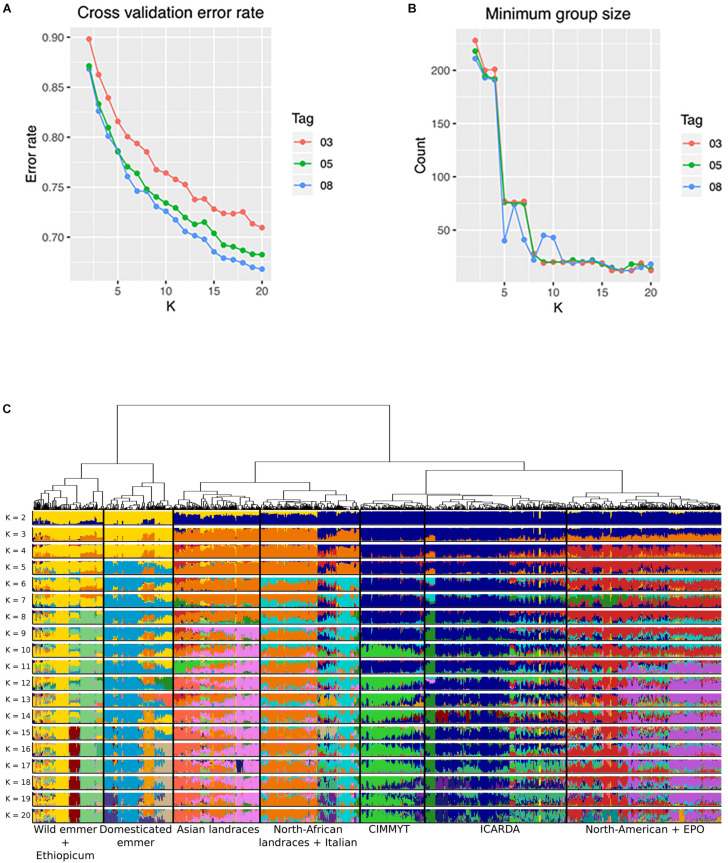
*ADMIXTURE’*s grouping statistics: **(A)** cross validation error rate, and **(B)** minimum group size, from *k* = 2 to *k* = 20 for three LD pruned SNP datasets (*r*^2^ = 0.3, *r*^2^ = 0.5, *r*^2^ = 0.8); **(C)** population structure of the GDP collection based on Ward’s clustering and *ADMIXTURE* (SNP dataset at *r*^2^ = 0.5); membership from *k* = 2 to *k* = 20.

Further pairwise comparisons were carried out for breeding groups that contributed more than 30 entries to the GDP (Figure 4). This investigation included modern *T. durum* genotypes from CIMMYT, ICARDA, Italy, France and North America, for a total of 10 pairwise comparisons. In total, 126 PSW clusters were identified (Supplementary Table S8), 59 of them supported by both indices, 40 based on *DRI* only, and 28 by *Fst* only. PSW cluster size ranged between 11 and 468 Mbp, with an average of 45.7 Mbp, and most clusters (81%) extending for less than 50 Mbp. Clusters were found in two or more comparisons (54), and only five were pair-specific. For 19 clusters a possible correspondence with a putative candidate gene could be proposed. The North American breeding group had the lowest number of PSW clusters (79), followed by CIMMYT with 88 clusters and the French breeding program with 100 PSW clusters. ICARDA and the Italian breeding programs had the highest numbers, 105 and 110, respectively. Considering pair-specific PSW clusters, CIMMYT and French groups showed the lowest number of specific PSW clusters (9), while Italy and ICARDA presented 12 and 11, respectively, and North America showed the highest number of specific PSW clusters (18).

### GDP Stratification Analysis

Population stratification was conducted based on both Ward’s clustering and admixture sub-population membership from *k* = 2 up to *k* = 20 based on the SNP dataset pruned at *r*^2^ = 0.5. Results of these analysis are shown in Figure 4C while Supplementary Table S9 reports sub-population memberships for each genotype and *K* value based on the two analyses. Applying SNP pruning with *r*^2^ = 0.8 outperformed the other two in terms of cross-validated group assignment (Figure 4A), although pruning at *r*^2^ = 0.5 provided comparable results. Grouping statistics, in particular the minimum group size (Figure 4B), stabilized at *k* > 11, despite the fact that cross-validated assignment error steadily decreased at higher *k* values (Figure 4A) and meaningful differences were still observed up to *k* values of 20. At *k* = 2, most accessions of *T. turgidum* spp. *dicoccum* (98%), *dicoccoides* (98%), *carthlicum* (92%) and *turgidum* (77%) clustered together (reported as dark yellow *Q* membership bars in Figure 4C), separated from all the durum wheat entries (reported as dark blue *Q* membership bars in Figure 4C). Notably, a small group of 33 (4%) of landraces from Ethiopia and the Arabian Peninsula clustered in the former group, showing appreciable genetic kinship with emmer from the Fertile Crescent. At *k* = 5, the emmer group was split in two main branches, one grouping wild emmer together with European and Fertile Crescent domesticated emmers, and the second having domesticated emmers from the Fertile Crescent together with Ethiopian durum and *T. turgidum* ssp. *carthlicum* entries. At *k* = 20, emmer accessions were further split between central Asian domesticated emmer (subp. 11), European domesticated emmer (subp. 12) and wild emmer (subp. 13).

At *k* = 2, the second mega-cluster included most *T. turgidum* ssp. *durum* (96%), *T. turgidum* ssp. *turanicum* and most of *T. turgidum* ssp. *polonicum* (67%). Separation between durum *modern* and *landraces* started at *k* = 3. At *k* = 6, durum landraces and primitive tetraploids were split into two main groups: Asian and North African landraces. Further meaningful landrace sub-groups were split at higher *k* values. The group including mainly Ethiopian accessions was split in two sub-groups: the first one contained accessions of *T. turgidum* spp. *carthlicum*, *polonicum* and *durum* landraces, while the second one was mainly *T. turgidum* ssp. *dicoccum* accessions, which might represent the founder group of Ethiopian durums.

Durum landraces and primitive tetraploids were grouped into subpopulations as follows: Central Mediterranean landraces (subp. 5), a mixed group of other Mediterranean landraces and old Italian cultivars such as the breeding germplasm founder Cappelli, and (subp. 6) more recent Italian cultivars directly related to landraces (subp. 7), Ethiopian durum landraces and emmers plus *T. turgidum* ssp. *carthlicum* (subp. 8), Central Asia durum landraces and all *T. turgidum* ssp. *turanicum* (subp. 10). Notably, sub-population 9 included a group of ICARDA founder cultivars belonging to the Om Rabi set, which were derived from crossing the Syrian landrace Haurani to the CIMMYT cultivar Jori (Kabbaj et al., 2017).

The modern durum germplasm was first split at *k* = 4 separating photoperiod sensitive accessions from northern countries (North America, France, Austria and the EPO entries) and Mediterranean-adapted photoperiod insensitive accessions. *K* = 10 was the minimum *k* value at which both Ward’s clustering and *ADMIXTURE* clearly separated the modern durum entries originating from the two main CGIAR (CIMMYT and ICARDA) breeding programs. At *k* = 13, modern durum entries were already divided in four sub-sets corresponding to French origin and EPO (subp. 1), CIMMYT (subp. 2), ICARDA (subp. 3), North American and Austrian (subp. 4). At *k* = 18 the group containing mainly CIMMYT durum wheat modern lines was further split in three sub-groups: the first one contained CIMMYT and other modern lines with different origins, the second one included CIMMYT and Egyptian germplasm, and the third one only modern germplasm from the Mediterranean countries. Only at *k* = 20 was the EPO set split into two groups.

**FIGURE 5:**
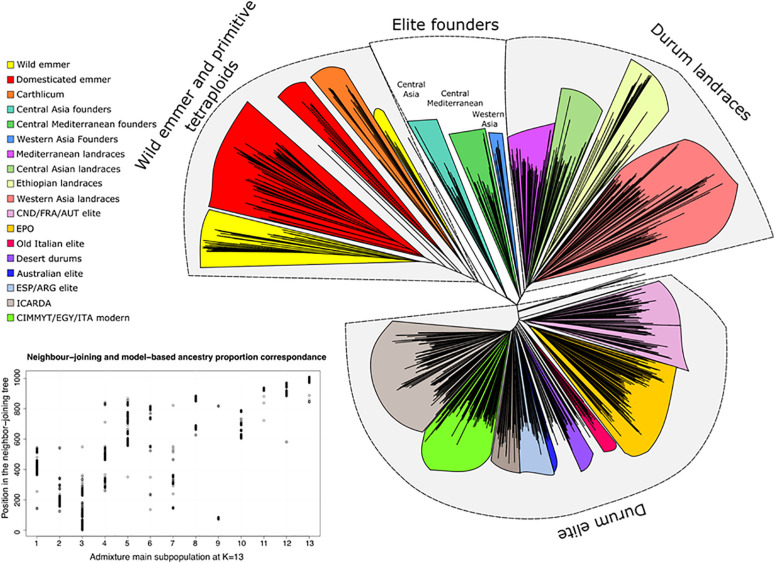
Neighbor joining tree of the GDP collection and comparison between NJ and ADMIXTURE model-based ancestry grouping methods. Details on accessions included in each clade are reported in Supplementary Table S9.

The GDP phylogenetic tree estimated through Neighbor-Joining clustering for all accessions is reported in Figure 5 and Supplementary Table S9. Bootstrap values indicating branches’ consistency are reported in detail in Supplementary Figure S13. Overall, good correlation was observed between population stratification analysis performed through admixture and the position on the Neighbor-Joining tree. Three main branches were grouped: (*i*) wild and domesticated emmers and *T. turgidum* ssp. *carthlicum*, (*ii*) durum landraces including the founders of modern germplasm and (*iii*) modern durums. Among durum landraces, one of the two sub-branches included North African/Southern European landraces and pioneering durum cultivars obtained from landrace selection and landrace intercrossing, such as Senatore Cappelli (selection from a landrace) and Capeiti8 (cross between Cappelli and a Syrian landrace selection). The second group included durum landraces from West Asia including Haurani, well-known as the most widely cultivated landrace population in its area of origin, showing developmental and morphological traits relevant for adaptation to low water availability and high temperatures, widely exploited by the ICARDA durum program since its inception (Elings and Nachit, 1991; Pagnotta et al., 2005). Another small group of interest is that composed of Central-Asian durum landraces that were included phylogenetically within the emmer clade. This group was found to lie between the main emmer clades and the modern durum, supporting a possible role of its members as founders of the Northern breeding programs (Paulsen and Shroyer, 2008).

## Discussion

### Genetic Diversity and Population Structure in GDP and Breeding Groups

The GDP builds on several studies that have investigated the diversity and phylogeny of durum wheat by assembling these into one panel. The two-step approach deployed here started by gathering entries representing nearly all genetic diversity studies ever conducted for durum wheat within the DWRC. In the second step, 1,011 entries were selected from the DWRC to capture most of this diversity (94–97%), with the strongest reduction affecting some rare alleles.

In the GDP, the mean PIC values of 0.27 for *landraces* and 0.28 for *modern* lines and ranging from 0.09 to 0.38 (Table 2B) indicated a generally higher or similar level of genetic diversity captured within the GDP compared to previously studied collections. Recent studies reported PIC values of 0.26 for durum modern germplasm (Chao et al., 2017), 0.19 for a set of both landraces and modern lines (Ren et al., 2012), and 0.18 in a collection of 168 durum wheat accessions of different origins (Roncallo et al., 2019). Analogously, AMOVA on clusters within GDP based on geography and breeding program of origin showed that only 13% of the total genetic variance could be captured among groups, while most diversity remained among individuals within clusters. These results concur with those reported by Soriano et al. (2016) with 172 landraces from 21 countries, by Roncallo et al. (2019) with a panel of 168 durum accessions and by N’Diaye et al. (2018) with a panel of Canadian durum cultivars where only 10% of variation was captured among groups. Other studies considering similar panels reported capturing over 30% of the total genetic variance by clustering germplasm based on kinship matrix, but using relatively higher *k* values (Kabbaj et al., 2017; Robbana et al., 2019). Our study aimed primarily at evaluating the historical diversity based on passport information, rather than on clusters derived from population structure. It is therefore evident that the passport information alone, while of great historical interest, is unable to capture the true genetic diversity of durum wheat worldwide. AMOVA on stratified groups may reveal much more variance among sub-populations, as indeed reported by other authors (Kabbaj et al., 2017; Roncallo et al., 2019). The moderate diversification among breeding groups (11.67% of the total variance) and very little among decades of release (2.95% of the total variance) revealed by AMOVA on the 473 modern durum wheat accessions (Tables 2D,E) was probably due to the wide and frequent exchange of parents among durum breeders worldwide. This was clearly evidenced in the Italian breeding programs, characterized by an overall higher level of diversity and lower differentiation against most of the other breeding programs, thus reflecting the necessity to breed for the many different agro-ecological zones that exist in Italy (Fischer et al., 2012). Overall, the results presented here suggest that good genetic diversity remains available within the breeding groups for direct exploitation, and there is even greater potential when considering exchanges between breeding groups.

The EPO is an evolutionary durum wheat pre-breeding population obtained through initial crossing of modern French varieties with various tetraploid wheat subspecies (David et al., 2014). When compared to *landraces* and *modern* durum lines, EPO lines showed the same level of genetic diversity in terms of mean number of pairwise differences and expected heterozygosity of *modern* lines, indicating that the genetic background of EPO lines is relatively homogeneous while being enriched in exotic alleles.

Substantial agreement between NJ, ADMIXTURE and Ward’s clustering indicated a complex, still well-defined stratification of the population, driven by historical, geographical and environmental factors. Phylogenetic analysis (Figure 5) highlighted three well-defined landrace groups of geographically distinct origin, holding a pivotal role as founders of different breeding programs. These included landraces from North Africa, West Asia and Central Asia as founders of modern breeding, in particular of ICARDA and Italy (Kabbaj et al., 2017; Soriano et al., 2018), while Central Asian landraces have played a critical role in the foundation of the North American modern durum germplasm via the early introduction from Russia and Turkey by Mennonite immigrants (Moon, 2008; Paulsen and Shroyer, 2008). The identification of these founders concurs with the results reported by Kabbaj et al. (2017), Maccaferri et al. (2019), and Taranto et al. (2020), supporting the validity of the phylogeny studies conducted for the GDP.

### Putative Signature of Selection Across the Breeding History and the Breeding Groups

Intense breeding in the past decades led to the development of superior cultivars for a broad range of edaphic environments. Current varieties exhibit increased yield potential, spike fertility, pasta quality and are resistant to widespread diseases such as rusts. The process of selection has evidently resulted in “signatures” being incorporated into the durum wheat genome, specific to each breeder’s targets and selection procedures, as well as shared preferences across breeding programs. The large set of *unique* alleles in the germplasm of historical breeding groups from Central Europe, Central Asia and Italy appear as a function of the longer effort to improve adaptation compared to more recent breeding groups. The large set of unique alleles, a high proportion of which were rare in Central Europe (58%) and Italy (99%), is consistent with extended selection for a particular environment. Studies aiming to describe allele fixation and genetic diversity are of great importance to guide breeders in planning their crosses and introgressions (Kabbaj et al., 2017; Taranto et al., 2020). In this regard, unique alleles can be seen as strategic targets for capturing exploitable genetic variability when linked to important traits.

The influence of selection on the genome was reflected in the diversity reduction index (*DRI*) and *F*_*st*_ metrics. Overall putative selection signals were found throughout the entire genome, including the centromeric regions. The average signal size of 50 Mbp suggested strong selection pressure. Several PSW clusters identified in this study co-located with known loci relevant to durum wheat breeding, thus demonstrating the predictive validity of the genome-wide search method. Expected signals associated with the transition from *landraces* to *modern* were related to the control of traits strongly selected in the post Green Revolution period causing the almost complete fixation of such loci in the *modern* subpopulations. As an example, *Cls-chr4B.2* included the widely used *Rht1-B* (Khush, 2001; Evenson and Gollin, 2003; Borojevic and Borojevic, 2005). This locus has also been identified as a putative signal of selection when comparing the ‘70–’80 and ‘81–’90 decades (*Cls-chr4B.1*, Supplementary Table S7) as well as when contrasting North American germplasm (tall cultivars) vs. Italy/France (semi-dwarf), and ICARDA (mix tall and semi-dwarf) vs. Italy (all semi-dwarf) breeding programs. Phenology is also a trait under strong and constant selection pressure, supported by the PSW cluster in the *landraces* vs. *modern* germplasm (Supplementary Table S6) that co-located with the photoperiod insensitive gene *Ppd-A1* (Beales et al., 2007; Maccaferri et al., 2008; Wilhelm et al., 2009; Bentley et al., 2011). The signal marked the transition from landraces to modern cultivars since the photoperiod insensitive allele was widely and positively selected, as already reported by Motzo and Giunta (2007). Following the Green Revolution, selection for photoperiod insensitivity continued as shown by the inclusion of both *PPD* homeologs on chromosomes 2A and 2B in cluster signals. PSW signals for the *Ppd-A1* and *Ppd-B1* regions were identified from comparisons of the Italian, French and ICARDA breeding groups vs. CIMMYT and North America groups, respectively (clusters *Cls_clv-chr2A.1* and *Cls_clv-chr2A.1*, *Cls-chr2B.1;* Supplementary Table S8), indicating a generalized selection strategy to fine tune the photoperiod insensitive alleles to match the ideal phenology for the targeted environment (Maccaferri et al., 2008).

Another important class of genes known to have undergone strong selective pressure in bread wheat are the *VRN*. In contrast to *PPD*, the PSW signal for *VRN* loci was much weaker in the GDP. For instance, no PSW cluster included *Vrn1*-5A (Yan et al., 2003), while *Vrn3-*7A (Yan et al., 2006) generated PSW signals in both A and B sub-genomes. For example, *Cls-chr7A.4* was identified in the North American group vs. ICARDA, CIMMYT and Italy; *Cls-chr7A.5* was identified for the comparisons of CIMMYT vs. ICARDA and Italy; and *Cls-chr7B.1* corresponded to *Vrn3-7B* for the comparisons of CIMMYT vs. France and Italy (Supplementary Table S8). Mild vernalization requirements are still present in *modern* cultivars for the Mediterranean areas where wheat is cultivated as a fall-sown cereal, and distinctions at these loci might depend on the breeder’s target of extending or reducing the overall cycle in different agro-ecologies. Lastly, among the earliness *per se* genes, *ELF3-A1* (Zikhali et al., 2016) appears the most likely candidate for the PSW cluster *Cls-chr1A.8*, which differentiated both France and North America *modern* germplasm when comparing ICARDA and Italy (Supplementary Table S8).

PSW clusters could also be related to selection for increased spike fertility and grain yield potential, particularly in the *landrace* to *modern* comparisons (Supplementary Table S6). This is the case of *Cls-chr3B.2* and *Cls-chr7A.2* whose intervals include the determinant of grain weight identified in bread wheat *TaCKX6* (cytokinin oxidase/dehydrogenase, Zhang et al., 2012) and *TaTGW-7A* (Hu et al., 2016), respectively. Additionally, *Cls-chr2A.4* and *Cls-chr2B.3* overlapped with the recently cloned gene related to floret fertility *GNI-A1* (Sakuma et al., 2019), while in some comparisons among breeding groups (Supplementary Table S8) a PSW cluster (*Cls-chr2A.3*) overlaps with *TaSus2* (sucrose synthase), a main driver of starch accumulation in wheat found to be associated with strong changes in haplotype frequency in bread wheat (Hou et al., 2014). Considering nitrogen metabolism and grain protein content, an important quality trait for durum wheat, the *landraces* vs. *modern* contrast co-located *Cls-chr2A.5* and *Cls-chr2B.5* with genes encoding for glutamine synthase *GS2-2A* and *GS2-2B* (Supplementary Table S8). Both these genes play a key role in high protein content (Gadaleta et al., 2011). Clusters could be related to selection for quality of grain proteins as shown by *Cls-chr1B.4* and *Cls-chr6A.1* overlapping with genes for glutenins (*Glu-B1*, Xu et al., 2008) and gliadins (*Gli-6A*, Gu et al., 2004), respectively. In particular, *Cls-chr6A.1* was detected for *landraces* vs. *modern* and for three breeding programs pairwise comparisons (i.e., ICARDA, CIMMYT and Italy vs. North America and France) (Supplementary Tables S6, S9), while *Cls-chr1A.1* was identified in three decade pairwise comparisons and in ICARDA vs. CIMMYT (Supplementary Tables S7, S9). The co-localization between PSW clusters and glutenin and gliadin alleles is not unexpected given the influence of these genes on pasta quality, which is a major target of selection. Convincingly, three chromosomes, 1A, 1B, and 6A, involved in seed storage proteins were represented in the PSW clusters: *Cls-chr1A.1* (PSW found for decade and breeding program pairwise comparisons, co-locating with *Glu-A3* and gliadins*), Cls-chr1B.4 (Glu-B1), Cls-chr6A.1*, *Cls-chr6A.2* and *Cls-chr6A.3*, with the last three PSW partially overlapping and co-locating with *Gli-6A* (Supplementary Tables S7, S9).

Lastly, presence of gene candidates was observed for three strong PSW clusters that occurred in chromosome 7B (*Cls-chr7B.3*, centromeric and *Cls-chr7B.12*, distal) and in chromosome 5B (*Cls-chr5B.5*) and that are putatively related to grain quality. The two signals in chromosome 7B were associated to a strong QTL for grain yellow pigment content (reviewed in Colasuonno et al., 2019). The phytoene synthase, *Psy-B1*, a major gene responsible for yellow pigment content in the wheat grain and a common target of modern durum breeding for semolina color is a strong candidate (Pozniak et al., 2007). A signal for this locus emerged from the comparison of *landraces* vs. *modern* lines and North America (*Cls-chr7B.12*) vs. French and ICARDA breeding groups (Supplementary Tables S6, S9). The signal also appeared for three decade pairwise comparisons (Supplementary Table S7). suggesting a common historical selection for yellowness based on a number of co-located QTL clusters (Roncallo et al., 2012; Giraldo et al., 2016; Colasuonno et al., 2019) associated to specific *Psy-B1* alleles (reviewed in Colasuonno et al., 2019).

A recent study Taranto et al. (2020), aiming to define PSW among Italian cultivars and landraces also identified several of the selection sweeps proposed here, including the major loci controlling phenology and quality characteristics.

In summary, the report of PSW clusters in this manuscript is a first attempt to carry out such analysis across breeding programs from different countries. Although the causative genes of the PSW clusters remain to be verified, several plausible candidates have been proposed. The GDP provides then an unprecedented opportunity for international collaborations to more effectively harness and exploit the diversity identified here.

## Conclusion

In the present study, a very large and diverse durum wheat panel referred to as the GDP has been assembled and made publicly available to drive further discovery and deployment of beneficial alleles. The GDP is maintained and distributed by ICARDA Genbank^5^ under Terms and Conditions of SMTA. The genotypic datasets (both raw data and upon quality filtering and imputing) can be found in the online repositories GrainGenes (see text footnote 3), and T3/Wheat (see text footnote 4). The genetic characterization of this panel increases the knowledge of genetic relationships and population structure of worldwide durum wheat, while facilitating the identification of the optimal sources of genetic diversity for a given target locus. The entire durum community is now empowered to use this panel to discover novel and useful alleles via GWAS. Finally, since the GDP is an open resource available to the whole community, the discovery of useful alleles can be immediately incorporated in breeding activities irrespective of the country or research group that makes the discovery. This is particularly true now that a number of genomic resources are available for wheat, including the reference sequence of the durum wheat genome (Maccaferri et al., 2019). We believe that this international effort is a great example of how a whole community can come together to support breeders in their efforts to adapt and develop more resilient durum wheat varieties able to withstand climate change and ensure a great future for this important crop.

## Data Availability Statement

The datasets presented in this study can be found in online repositories: GrainGenes https://wheat.pw.usda.gov/GG3/global_durum_genomic_resources, and T3/Wheat https://wheat.triticeaetoolbox.org/breeders_toolbox/protocol/158.

## Author Contributions

FB, RT, MM, LC, JA, KA, and SX designed this initiative. EM, DM, SC, SX, JF, and MH produced the genotypic data and all authors supported the genotyping. EM, GS, AM, FD, GP, MM, RT, LC, and FB analyzed the data. EM, GS, AM, MM, and FB developed the first draft. All authors reviewed and approved the final version of this manuscript.

## Conflict of Interest

The authors declare that the research was conducted in the absence of any commercial or financial relationships that could be construed as a potential conflict of interest. The reviewer MR declared a past co-authorship with several of the authors DM and FL to the handling editor.

## Footnotes

1 https://www.cwrdiversity.org/

2 www.wheatinitiative.org

3 https://wheat.pw.usda.gov/GG3/global_durum_genomic_resources

4 https://wheat.triticeaetoolbox.org/breeders_toolbox/protocol/158

5 http://indms.icarda.org/

## Supplementary Material

FIGURE S1 Population structure of the DWRC collection based on ADMIXTURE analysis.

FIGURE S2 Population structure of the DWRC collection based on bootstrapped Ward’s clustering.

FIGURE S3 Bootstrapped Ward’s clustering of the DWRC subgroup of *T. durum* cultivars, varieties and élite lines.

FIGURE S4 Bootstrapped Ward’s clustering of DWRC subgroup EPO.

FIGURE S5 Bootstrapped Ward’s clustering of DWRC subgroup of *T. durum* landraces.

FIGURE S6 Bootstrapped Ward’s clustering of DWRC subgroup of *T. dicoccum* accessions.

FIGURE S7 Bootstrapped Ward’s clustering of DWRC subgroup of *T. dicoccoides* accessions.

FIGURE S8 Bootstrapped Ward’s clustering of DWRC subgroup of *T. turgidum* subspecies *carthlicum, aethiopicum, polonicum, turanicum, turgidum.*

FIGURE S9 Sampling effect on genetic diversity between DWRC and GDP: correlation of diversity indexes between GDP and DWRC for Shannon-Wiener index, expected heterozygosity, evenness, and minor allele frequency.

FIGURE S10 Site frequency spectrum of loci in GDP and DWRC.

FIGURE S11 Distribution of the SNPs along the chromosome and inter SNP distances. **(A)** Average number of SNPs per classes of interlocus distances, across all chromosomes; **(B)** number of SNPs per each chromosome segment, from proximal (1) to distal (10) regions, mediated across all chromosomes; **(C)**: interlocus distances in each chromosome segment, from proximal (1) to distal regions (10), presented for all chromosomes combined.

FIGURE S12 Local LD of *T. durum* landraces and modern lines presented for each chromosome.

FIGURE S13 Bootstrap neighbor joining phylogenetic tree of GDP.

TABLE S1 List of private companies, institutions, international organizations which contributed tetraploid wheat germplasm to the initial DWRC.

TABLE S2 DWRC: **(A)** list of accessions constituting the DWRC; **(B)** scoring of DWRC accessions based on KASP marker set; **(C)** KASP markers list used for the DWRC genotyping.

TABLE S3 List of accession constituting the GDP, with passport data. The categories based on passport data used to classify accessions for the diversity analyses are also reported, as well as available data about flowering habit and allele status at some known genes (*Rht*, *Ppd*, *Cdu*, *Vrn*, etc.).

TABLE S4 Genetic distance matrix of the GDP.

TABLE S5 List of unique alleles within the breeding groups of the GDP.

TABLE S6 PSWs between *modern* and *landraces:* **(A)** list of clusters of PSWs with position on the Svevo reference genome, metrics detecting PSWs, and the candidate gene; **(B)** significant values for each metrics (*F*_*st*_, DRI) for each SNP sliding window.

TABLE S7 PSWs for modern, between different decades: **(A)** list of clusters of PSWs between different decades, with position on the Svevo reference genome, metrics detecting PSWs in each comparison, and the candidate gene; **(B)** significant values for each metrics (*F*_*st*_, DRI) for each SNP sliding window in each comparison.

TABLE S8 PSWs for *modern*, between different breeding groups: **(A)** list of clusters of PSWs between different breeding programs, with position on the Svevo reference genome, metrics detecting PSWs for each comparison, and the candidate gene; **(B)** significant values for each metrics (*F*_*st*_, DRI) for each SNP sliding window in each comparison.

TABLE S9 Stratification analysis of GDP: **(A)** grouping on the base of the main model-based ancestry estimation and neighbor joining tree position. Accessions are sorted on the base of their position on the NJ tree of Figure 5 and colors correspond to those of groups highlighted in the same Figure 5; **(B)** Ward’s clustering of GDP from K2 to K20; **(C)** membership value for each GDP accession at *K* = 13 based on ADMIXTURE ancestry estimation.
