# Supplementary material for: The Global Durum Wheat Panel (GDP): An International Platform to Identify and Exchange Beneficial Alleles
Source: Front Plant Sci. 2020 Dec 21;11:569905. doi: 10.3389/fpls.2020.569905 (PMC7779600; doi:10.3389/fpls.2020.569905)

Fig. S5. Distribution of the SNPs along the chromosome and inter SNP distances. **A**: average number of SNPs per classes of interlocus distances, across all chromosomes; **B**: number of SNPs per each chromosome segment, from proximal (1) to distal (10) regions, mediated across all chromosomes; **C**: interlocus distances in each chromosome segment, from proximal (1) to distal regions (10), presented for all chromosomes combined.

A.

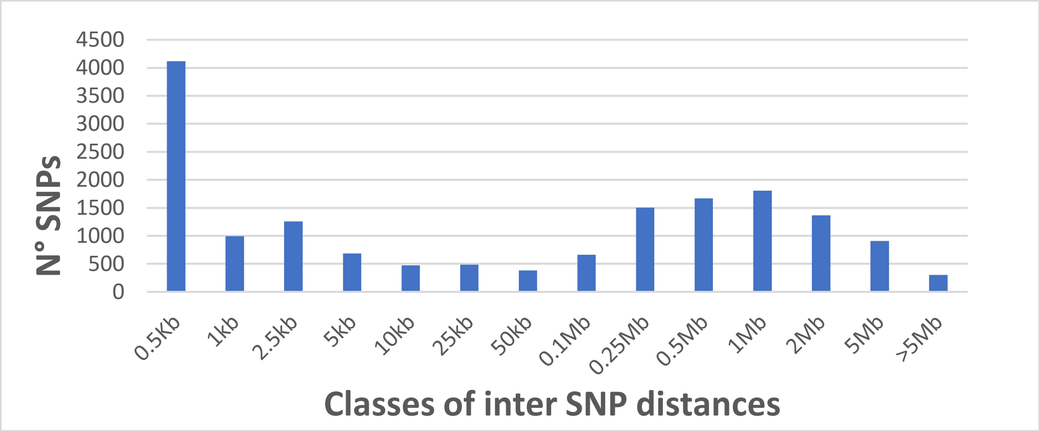

B

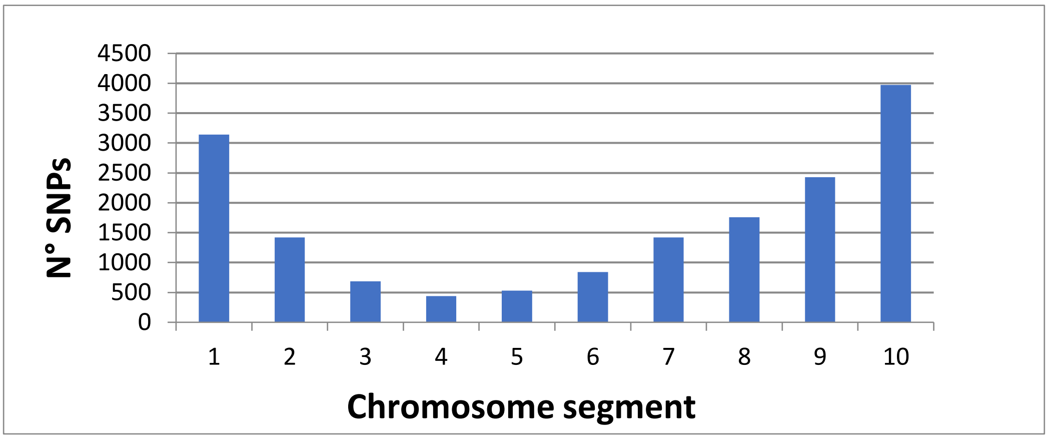

C

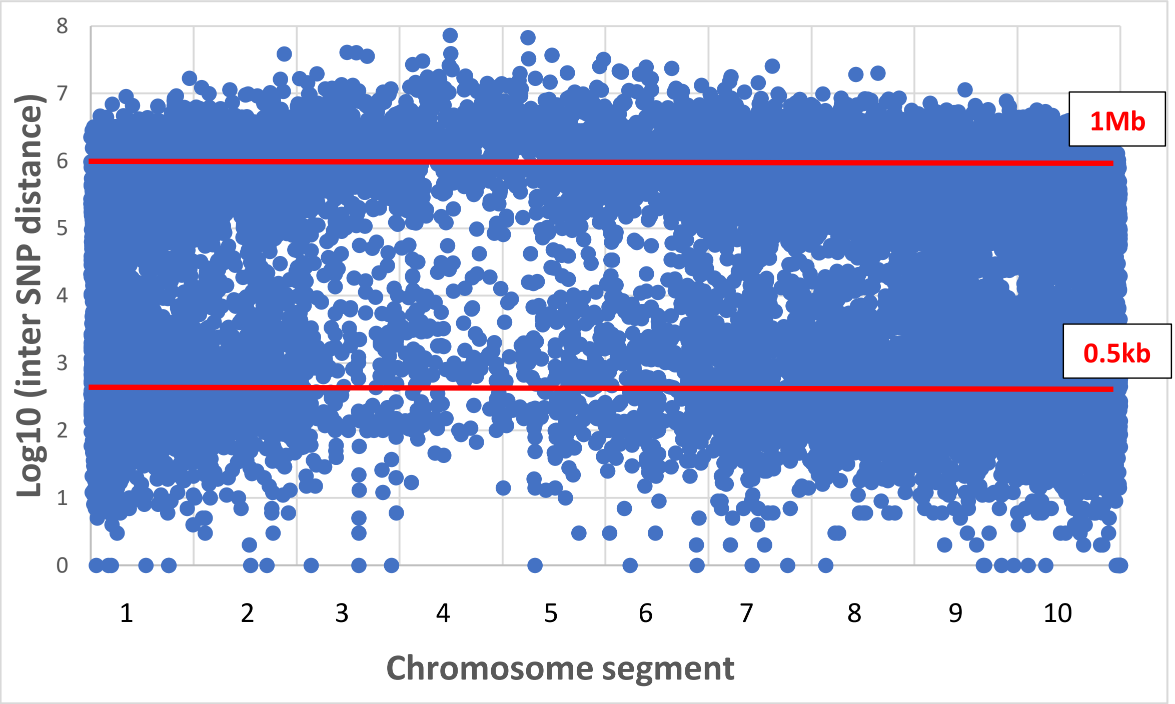

Supplement: FIGURE S1 — Population structure of the DWRC collection based on ADMIXTURE analysis. [file Data_Sheet_1.ZIP › Supplementary Fig S11.pdf]
