# Supplementary figures and images for: The Global Durum Wheat Panel (GDP): An International Platform to Identify and Exchange Beneficial Alleles
Source: Front Plant Sci. 2020 Dec 21;11:569905. doi: 10.3389/fpls.2020.569905 (PMC7779600; doi:10.3389/fpls.2020.569905)

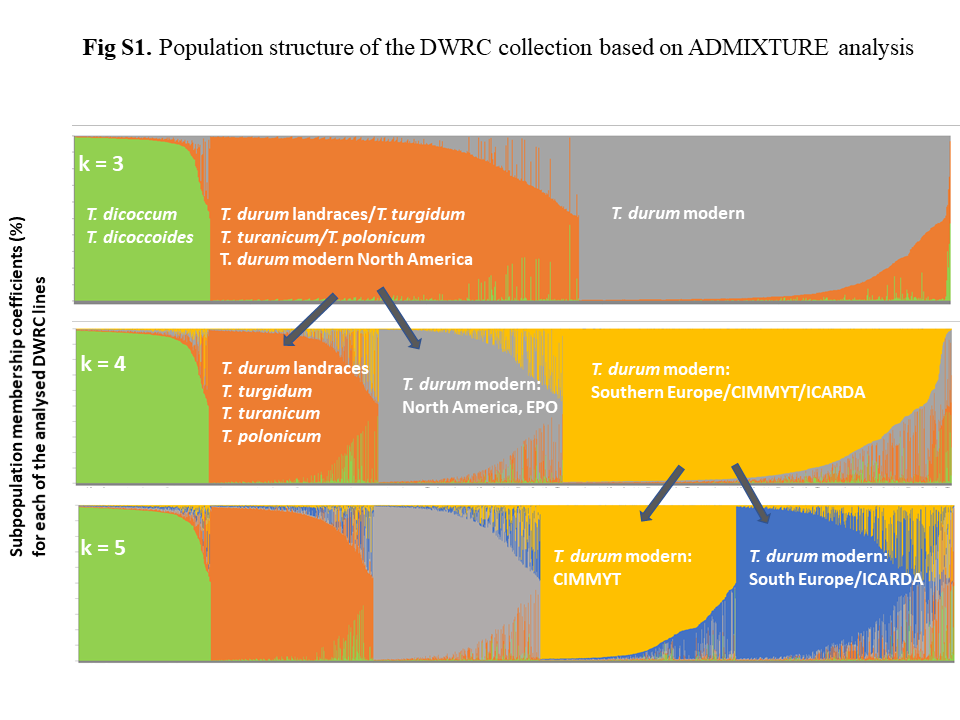

Supplement: FIGURE S1 — Population structure of the DWRC collection based on ADMIXTURE analysis. [file Data_Sheet_1.ZIP › Supplementary Fig S1.tif]

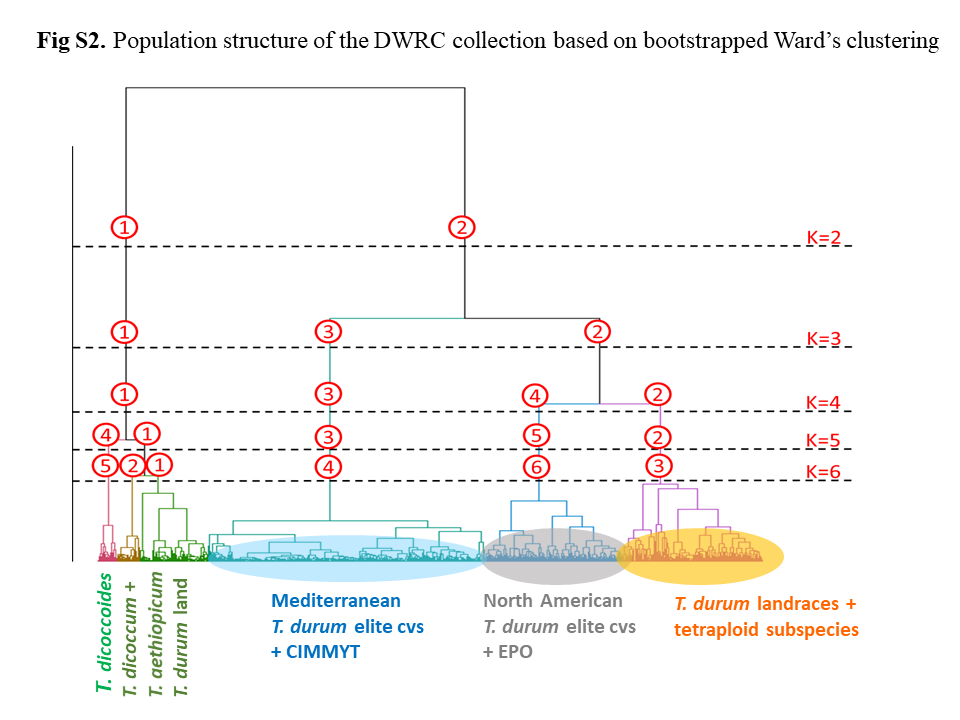

Supplement: FIGURE S1 — Population structure of the DWRC collection based on ADMIXTURE analysis. [file Data_Sheet_1.ZIP › Supplementary Fig S2.tif]

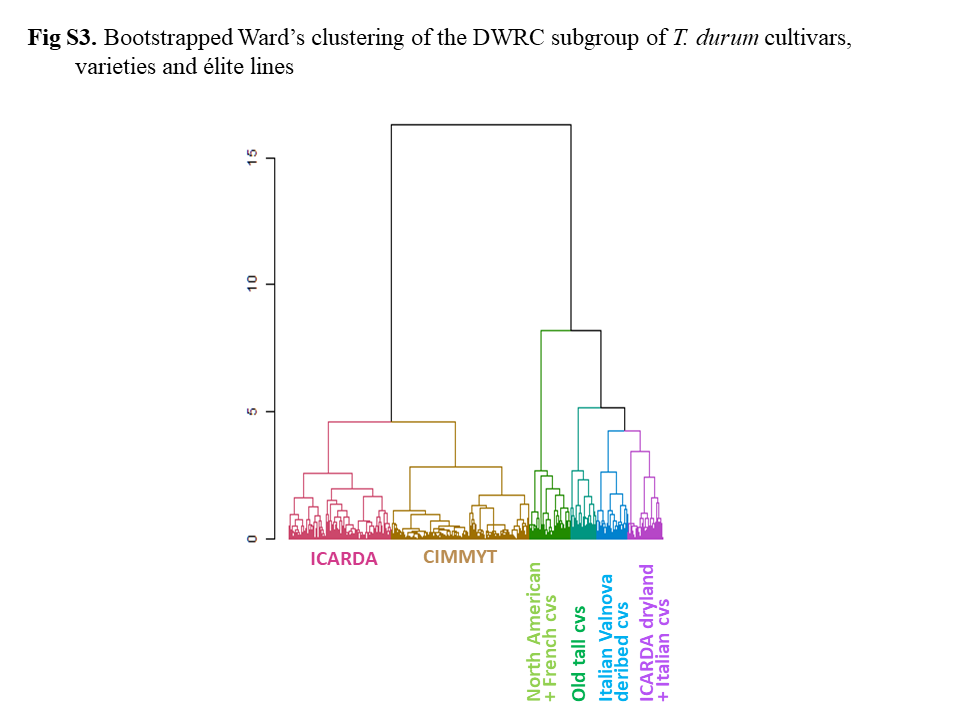

Supplement: FIGURE S1 — Population structure of the DWRC collection based on ADMIXTURE analysis. [file Data_Sheet_1.ZIP › Supplementary Fig S3.tif]

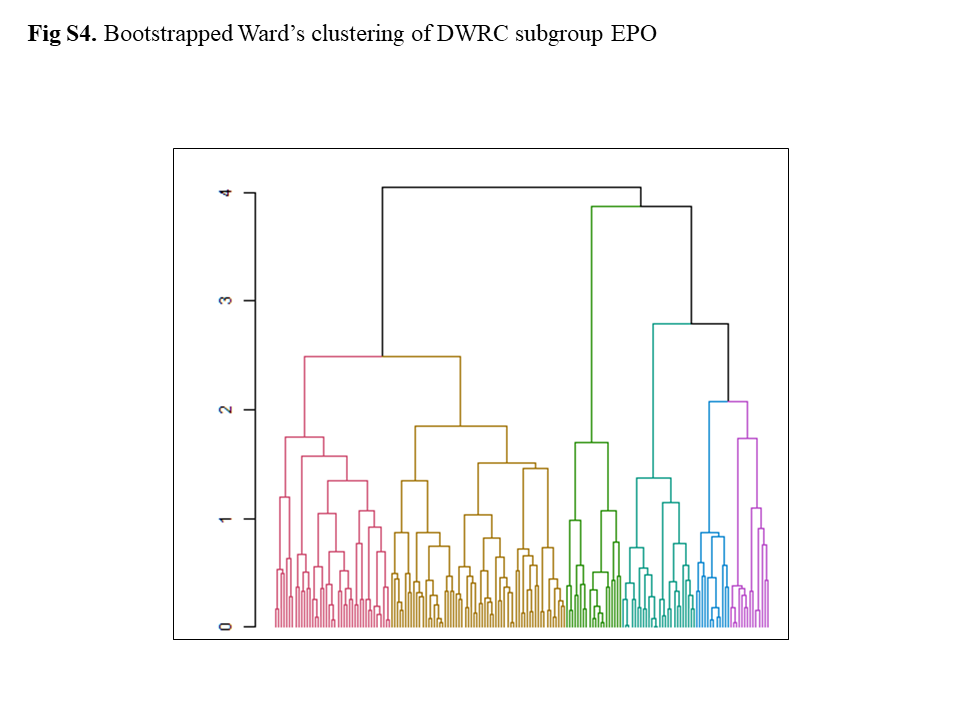

Supplement: FIGURE S1 — Population structure of the DWRC collection based on ADMIXTURE analysis. [file Data_Sheet_1.ZIP › Supplementary Fig S4.tif]

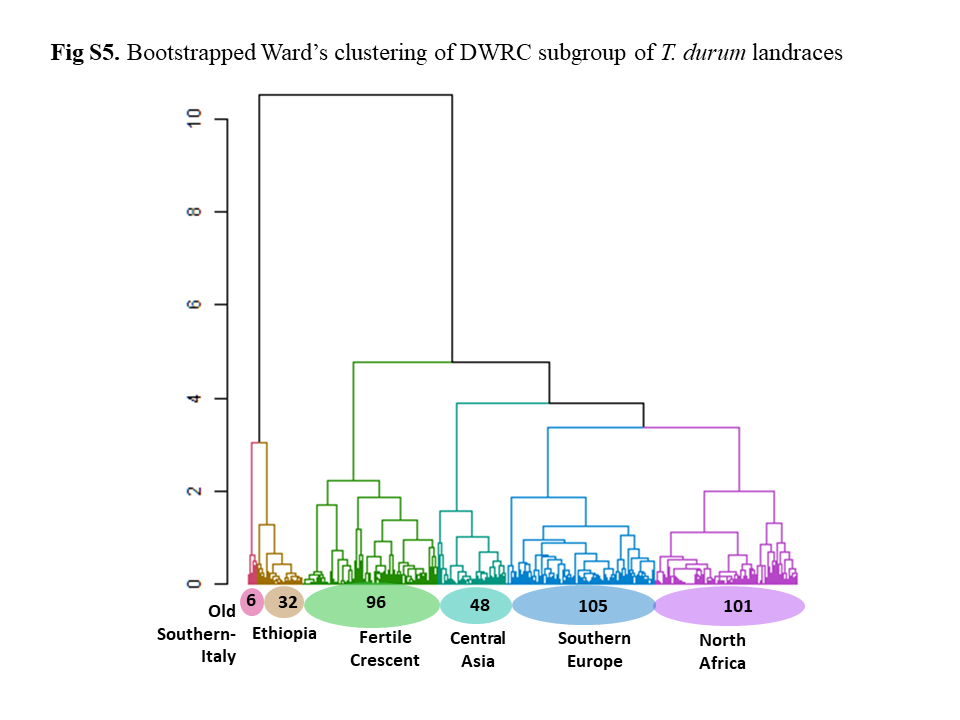

Supplement: FIGURE S1 — Population structure of the DWRC collection based on ADMIXTURE analysis. [file Data_Sheet_1.ZIP › Supplementary Fig S5.tif]

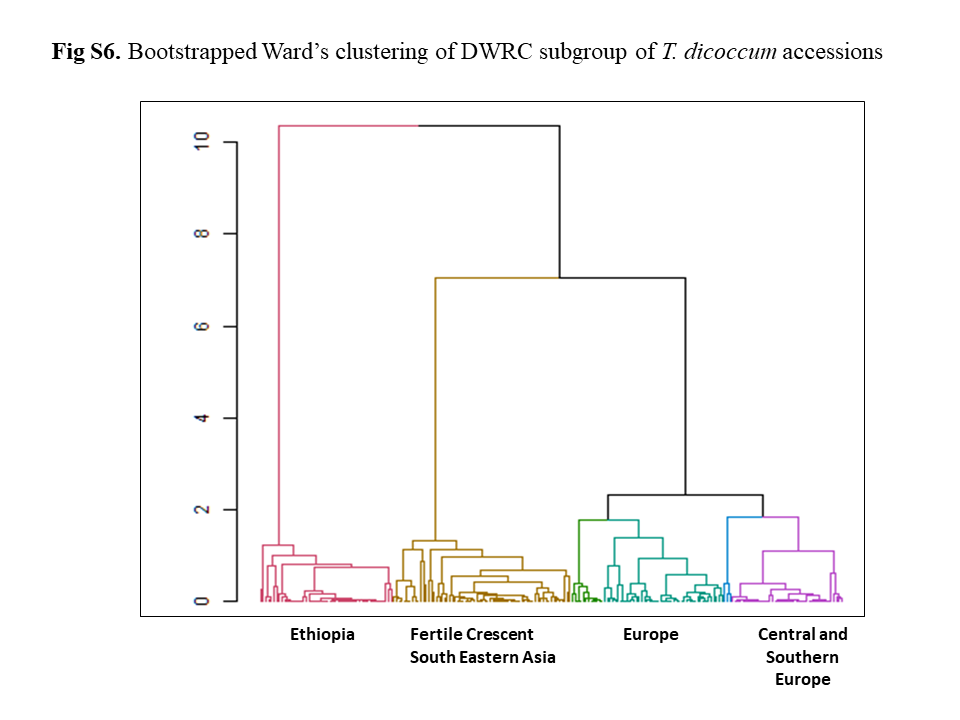

Supplement: FIGURE S1 — Population structure of the DWRC collection based on ADMIXTURE analysis. [file Data_Sheet_1.ZIP › Supplementary Fig S6.tif]

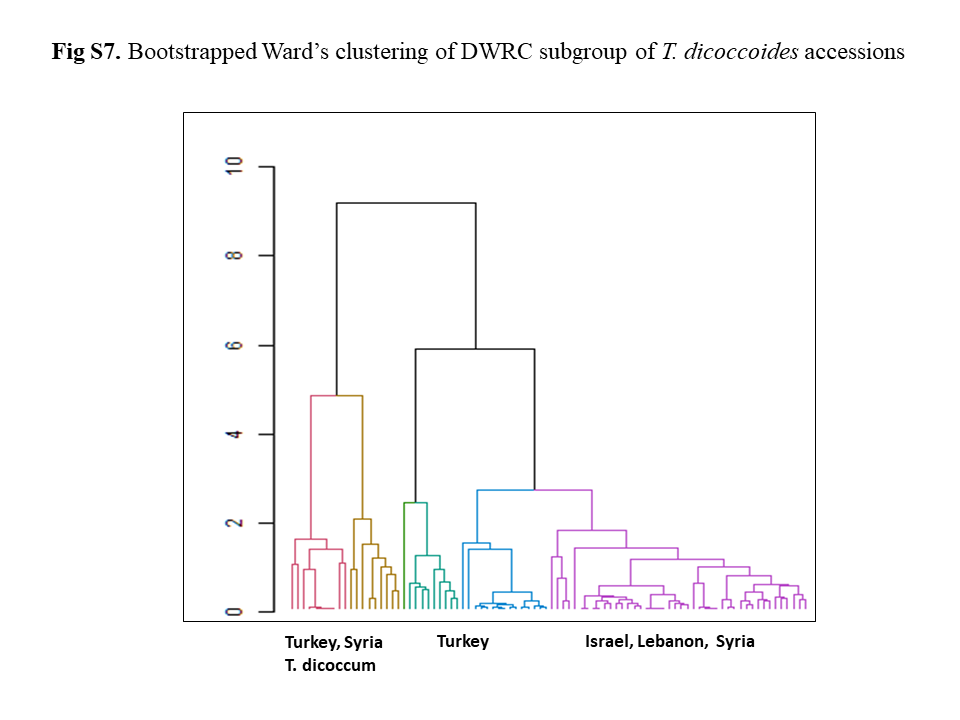

Supplement: FIGURE S1 — Population structure of the DWRC collection based on ADMIXTURE analysis. [file Data_Sheet_1.ZIP › Supplementary Fig S7.tif]

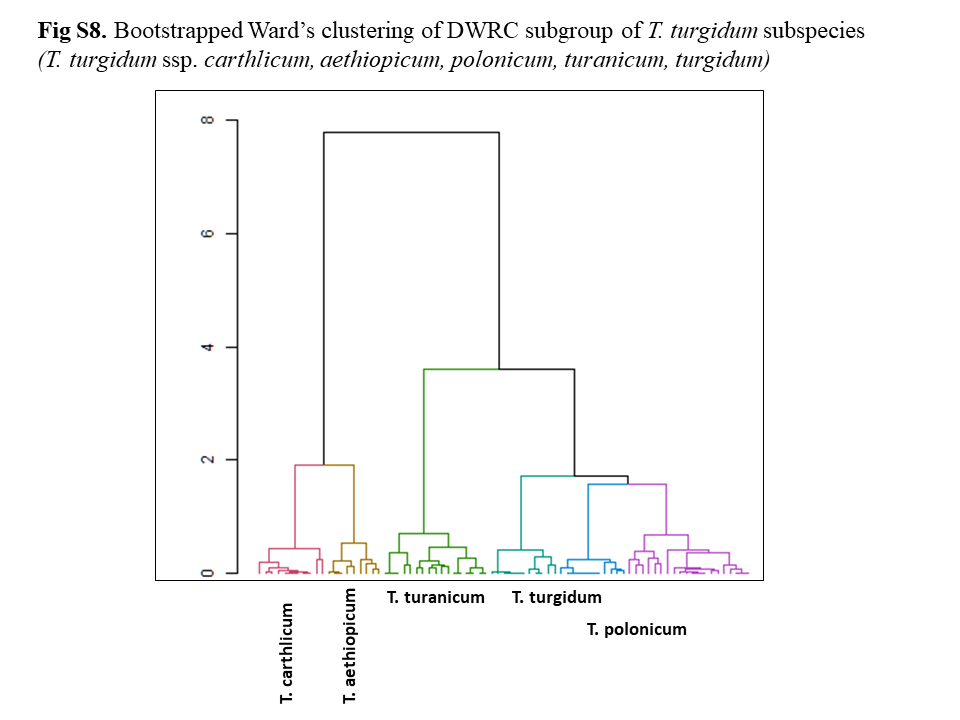

Supplement: FIGURE S1 — Population structure of the DWRC collection based on ADMIXTURE analysis. [file Data_Sheet_1.ZIP › Supplementary Fig S8.tif]

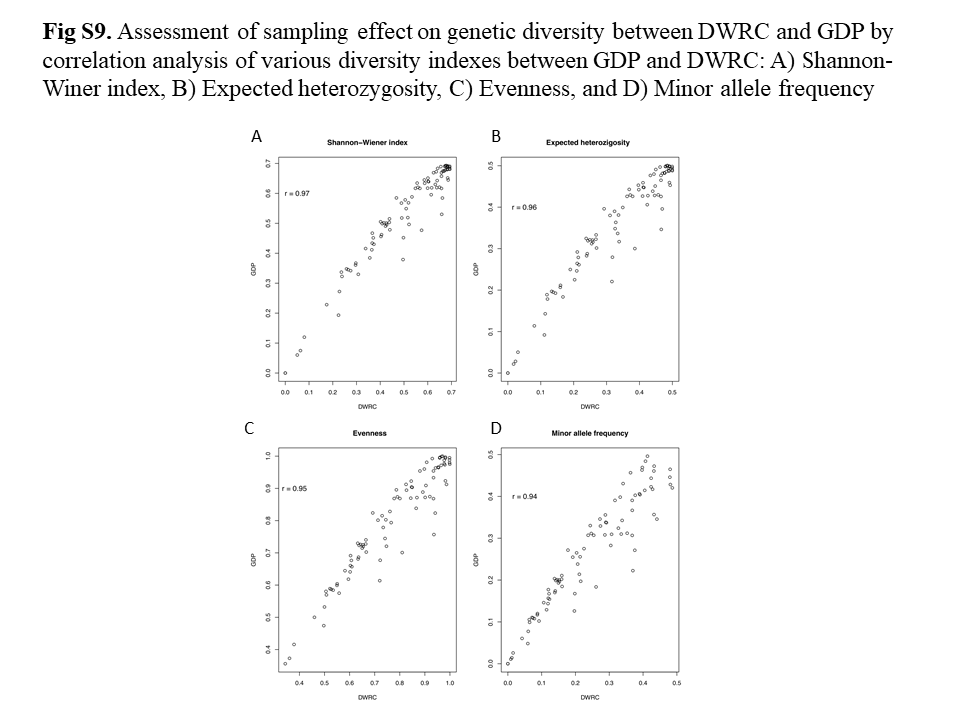

Supplement: FIGURE S1 — Population structure of the DWRC collection based on ADMIXTURE analysis. [file Data_Sheet_1.ZIP › Supplementary Fig S9.tif]

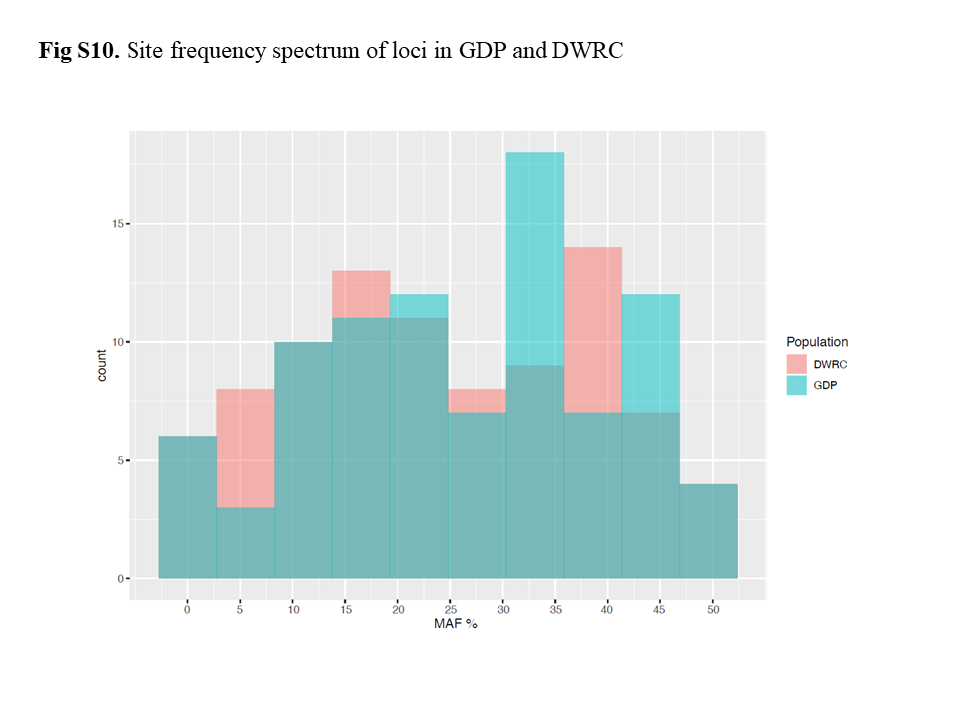

Supplement: FIGURE S1 — Population structure of the DWRC collection based on ADMIXTURE analysis. [file Data_Sheet_1.ZIP › Supplementary Fig S10.tif]

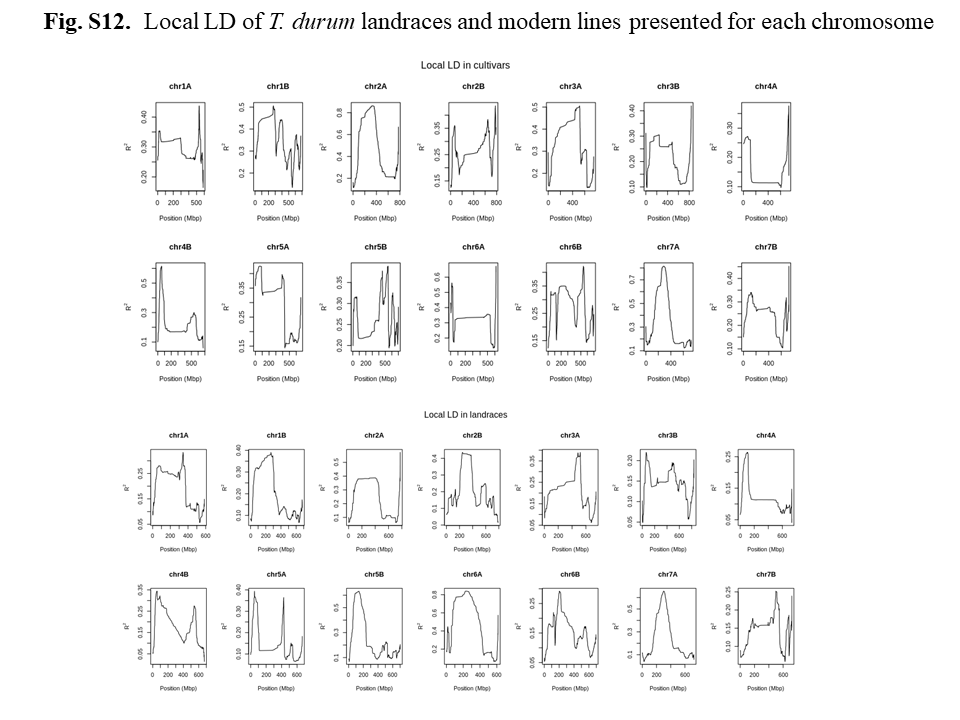

Supplement: FIGURE S1 — Population structure of the DWRC collection based on ADMIXTURE analysis. [file Data_Sheet_1.ZIP › Supplementary Fig S12.tif]

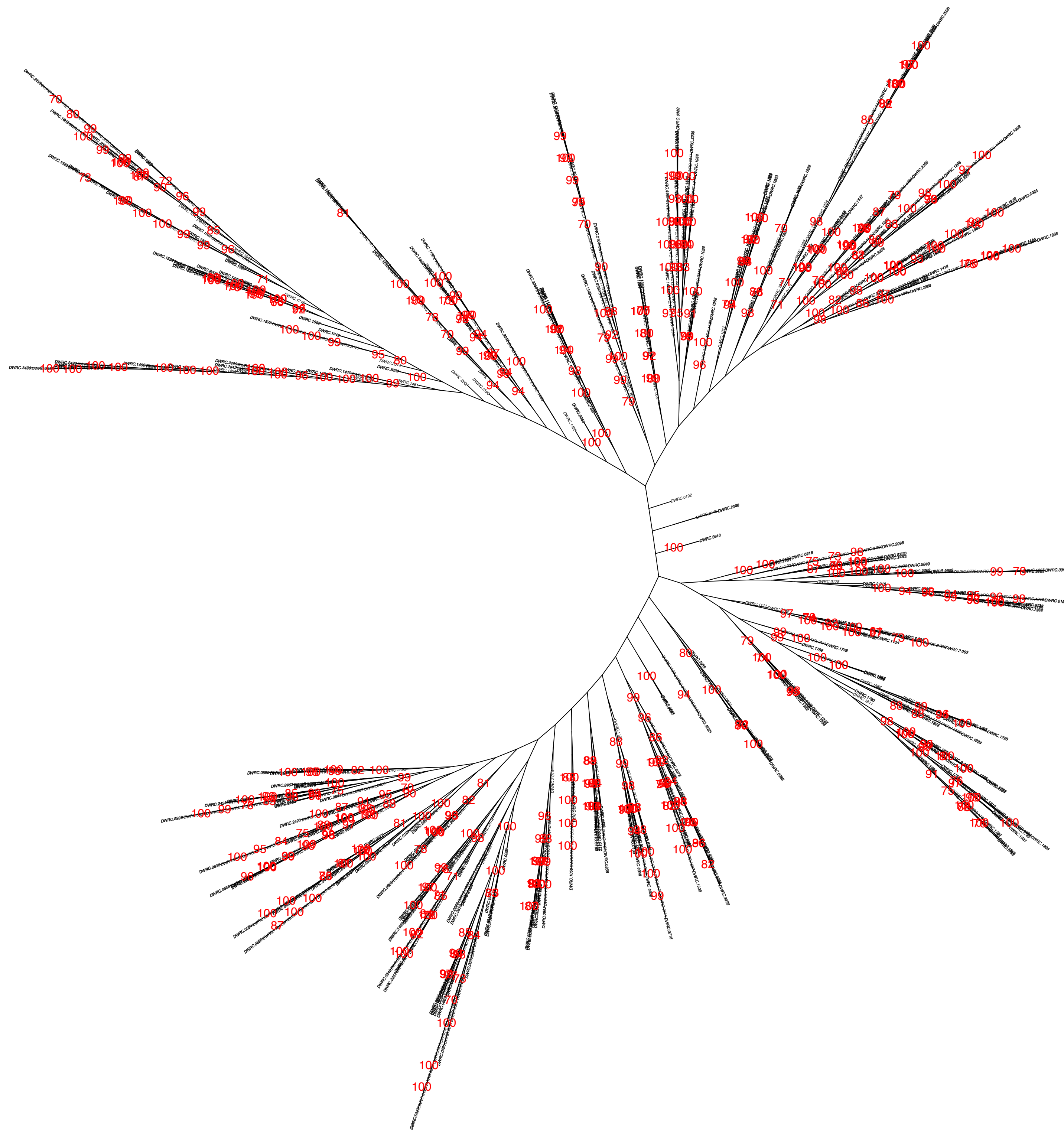

**Fig. S13.** Bootstrapped neighbor joining phylogenetic tree of the GDP

Supplement: FIGURE S1 — Population structure of the DWRC collection based on ADMIXTURE analysis. [file Data_Sheet_1.ZIP › Supplementary Fig S13.pdf]
