# Supplementary material for: The Global Durum Wheat Panel (GDP): An International Platform to Identify and Exchange Beneficial Alleles
Source: Front Plant Sci. 2020 Dec 21;11:569905. doi: 10.3389/fpls.2020.569905 (PMC7779600; doi:10.3389/fpls.2020.569905)
Supplement: TABLE S1 — List of private companies, institutions, international organizations which contributed tetraploid wheat germplasm to the initial DWRC. [file Data_Sheet_2.ZIP › Supplementary Table S1_Contributors of GDP accessions.docx]

**Supplementary Table S1**. List of Private Companies, Institutions, International Organizations which contributed tetraploid wheat germplasm to the initial DWRC.

| **Institution** | **Country** |
| --- | --- |
| Aca Semillas | Argentina |
| AGROVEGETAL | Spain |
| Biogranum | Serbia |
| BUCK SEMILLAS SA | Argentina |
| Limagrain Europe | France |
| Saatzucht-Donau | Austria |
| AAFC - Agriculture and Agri-Food Canada | Canada |
| Beni Swif University (Faculty of Agriculture) | Egypt |
| CNR-National Research Centre (IBBR) | Italy |
| CONICET- National Scientific and Technical Research Council (CERZOS) | Argentina |
| CREA-Council for Agricultural Research and Economics (Research Centre for Genomics and Bioinformatics) | Italy |
| CREA-Council for Agricultural Research and Economics (Research Centre for Cereal and Industrial Crops) | Italy |
| EIAR-Ethiopian Agricultural Research Insititute | Ethiopia |
| INRA- French National Institute for Agricultural Research (AGAP) | France |
| INTA- National Agricultural Technology Institute (CEI_Barrow) | Argentina |
| IPBB-Institute of Plant Biology and Biotechnology | Kazakhstan |
| IRTA - Institute of Agrifood Research and Technology | Spain |
| NAAS - National Academy of Agrarian Sciences of Ukraine (Plant Production Institute nd. a. V. Ya. Yuryev) | Ukraine |
| RIGLC- Research Institute of Grain and Legume Crops | Uzbekstan |
| University of Bari (DISAAT) | Italy |
| University of Boku (Department of Agrobiotechology) | Austria |
| University of Bologna (DISTAL) | Italy |
| USDA- United States Department of Agriculture (ARS) | USA |
| CIMMYT - International Maize and Wheat Improvement Center | Mexico |
| ICARDA - International Center for Agricultural Research in the Dry Areas | Morocco |
